# Mesentery AjFGF4–AjFGFR2–ERK pathway modulates intestinal regeneration via targeting cell cycle in echinoderms

**DOI:** 10.1111/cpr.13351

**Published:** 2022-10-20

**Authors:** Chuili Zeng, Ming Guo, Yangxi Xiang, Mingshan Song, Ke Xiao, Chenghua Li

**Affiliations:** ^1^ State Key Laboratory for Managing Biotic and Chemical Threats to the Quality and Safety of Agro‐Products Ningbo University Ningbo China; ^2^ Laboratory for Marine Fisheries Science and Food Production Processes Qingdao National Laboratory for Marine Science and Technology Qingdao China

## Abstract

**Objectives:**

The purpose of the study aims to understand the regeneration process and its cytology mechanism in economic echinoderms.

**Materials and Methods:**

The intestine regeneration process of *Apostichopus japonicus* was investigated by immunohistochemistry and the cell proliferation was detected by immunofluorescence and flow cytometry. Fibroblast growth factor 4 of *A. japonicus* (AjFGF4) was screened by RNA‐seq analysis and validated to regulate cell proliferation by siAjFGF4 and recombinant‐AjFGF4 treatment. The binding and co‐localization of AjFGF4 and AjFGFR2 were verified by Co‐IP, GST‐pull down, and immunofluorescence. Then, the AjFGF4‐AjFGFR2‐ERK‐cell cycle axis was examined by western blot, immunofluorescence, and flow cytometry techniques.

**Results:**

The mesentery was served as the epicenter of intestinal regeneration via activating cell proliferation and other cellular events. Mechanically, AjFGF4‐mediated cell proliferation was dependent on the binding to its receptor AjFGFR2, and then triggered the conserved ERK–MAPK pathway but not JNK and p38 pathway. The activated ERK–MAPK subsequently mediated the expression of cell cycle regulatory proteins of CDK2, Cyclin A, and Cyclin B to promote cell proliferation.

**Conclusions:**

We provide the first functional evidence that AjFGF4‐AjFGFR2‐ERK‐cell cycle axis mediated cell proliferation was the engine for mesentery‐derived intestine regeneration in echinoderms.

## Graphical abstract

Mesentery served as the epicenter of intestinal regeneration to form a new intestine mainly via cell proliferation. Fibroblast growth factor 4 (AjFGF4) was found to regulate cell proliferation by specifically binding to AjFGFR2, and then triggered the conserved ERK–MAPK pathway but not JNK and p38 pathway. The activated ERK–MAPK subsequently mediated the expression of cell cycle regulatory proteins of CDK2, Cyclin A, and Cyclin B to promote cell proliferation.

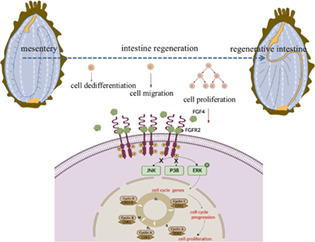

## 1 INTRODUCTION

Damage to organs or tissues by infection, trauma, aging, diseases, congenital defects, and other injuries causes organ malfunction and is life‐threatening under serious conditions.^1^ To maintain, improve, and restore organ functions after injury, one of the most exciting current biomedical research challenges is to decipher the molecular basis of organ regeneration, which is regulated by a series of dynamic and complex processes including the interplay among intracellular and extracellular signals, growth factors, cytokines, and other components.^2^ Thus, the explorations of the basic principles and the molecular underpinnings of regeneration have provided a rich biological resource and tools for understanding, building, or repairing complex body parts, which could bring us a step closer to regenerating human organs.^3^, ^4^, ^5^ However, the regeneration potentials present a heterogeneous distribution from the lowest to the highest phyla.^6^ Adult mammals, in particular humans, are not categorized as regeneration‐competent species, with a limited organ regenerative capacity following injury.^2^, ^4^ Whereas many lower vertebrates or invertebrates, such as amphibians, fish, and echinoderm possess extraordinary abilities to regenerate missing structures, which are used as models for regeneration studies in hopes of serving as a breakthrough in addressing regeneration studies in higher organisms. For instance, the research on heart regeneration of zebrafish and neonatal mouse is expected to inform the development of therapeutic strategies to treat heart disease.^7^, ^8^

The study of intestinal regeneration is expected to provide a reference for the treatment of intestine diseases, especially in some cases, intestinal tissue can be lost to various disorders and fails to support the needs of the organism. However, the intestine regeneration ability varies from species to species. For example, in widely studied mammals, the epithelial lining of the intestine is renewed continuously to repair local damage.^9^ By contrast, other lower invertebrates are capable of de novo morphogenesis of gastrointestinal organs. One of the most striking cases is the intestine regeneration of sea cucumbers, which possess an extraordinary capacity to regenerate their intestine at the whole‐organ scale after spontaneous evisceration and some species even could achieve this in less than 1‐week postevisceration.^10^ These features have made the evisceration and regeneration processes in sea cucumbers a suitable and attractive systematic model for studying intestine regeneration and further provide references to develop treatment options for intestine diseases.^11^ To date, the visceral regeneration has been documented in sufficient detail from four holothurian species belonging to two orders of Holothuriida (*Holothuria* [*Selenkothuria*] *glaberrima*) and Dendrochirotida (*Eupentacta fraudatrix* and *Cladolabes schmeltzii*).^12^, ^13^, ^14^ Morphological and molecular events of regeneration in them were studied in detail, morphogenesis was described on the cellular level, and various possible sources of gut regeneration were identified, which helps interpret molecular genetic data. However, the complete description from morphological observation to functional validation was scarcely investigated in certain species. Moreover, each of the holothurian species above has its own characteristics of regeneration, which allows for a more comprehensive exploration of intestinal regeneration research.^15^

Sea cucumber (*Apostichopus japonicus*) is an essential commercial Holothuroidea species in Asia aquaculture, and its annual worldwide trade has exceeded 15 billion US dollars. Like many other holothurians, *A. japonicus* has the capability to regenerate its lost digestive tract under suitable breeding conditions. However, such a spontaneous intestine remodeling process is an additional energy expenditure that diverts nutrients from growth and development, negatively affects body weight and reproduction, and sometimes even affects the function of the defense system, which is a bottleneck for the development of its commercial and subsistence fisheries.^16^ To date, most studies on intestinal regeneration of *A. japonicus* have focused on the morphological formation of the new intestine post‐self‐evisceration, while little is known about the cellular characteristics and molecular signaling mechanisms in the process of intestinal regeneration.^17^, ^18^ In the present study, we firstly investigated the process of intestine regeneration following autonomy by describing the spatial and temporal pattern of morphology and cellular events in *A. japonicus*. Impressively, we observed that the thickening of the free end of the mesentery was crucial to the formation of the intestine and such a process was always accompanied by a massive cell proliferation, which might supply additional cells necessary for the formation of new tissue. Then, RNA‐seq technology was conducted to identify the potential molecules for cell proliferation regulation in the process of intestinal regeneration, and a possible candidate gene fibroblast growth factor 4 (AjFGF4) was found significantly enriched during all the regeneration stages. Further assays confirmed that the AjFGF4‐mediated cell proliferation was facilitated by binding its receptor AjFGFR2, specifically activating the ERK–MAPK pathway and promoting the expression of cell cycle‐related protein (CDK2, Cyclin A, and Cyclin B) during intestine regeneration. Taken together, our work not only emphasized the importance of mesentery in intestinal regeneration and uncovered a previously unrecognized mechanism of the mesentery AjFGF4–AjFGFR2–ERK pathway modulates intestinal regeneration via targeting cell cycle in echinoderms, but also provides a reference for the exploration of internal organ regeneration in humans.

## 2 MATERIALS AND METHODS

### 2.1 Animals and treatment

Healthy sea cucumbers *A. japonicus* (average weight: 100 ± 5 g) were collected from a farm in Dalian, Liaoning province, China. They were quarantined in oxygen‐supplying natural seawater (salinity 28, temperature at 11°C), and fed daily with mixed feed for 1 week before treatment. After acclimation, these organisms were treated to induce evisceration by intra‐coelomic injection of 0.35 M KCl (3–5 ml) and left in seawater aquaria to undergo intestinal regeneration.^18^ Un‐eviscerated animals were kept under the same conditions and served as the controls.

### 2.2 Histomorphology observation

The regeneration tissues including mesentery and/or intestinal rudiment were sampled at 2, 7, 12, 20, and 28 days postevisceration (dpe) treatment. The regeneration tissues of healthy sea cucumbers without any treatment were also sampled and served as the control. These sampled tissues were fixed in Bouin's fluid for 24 h and dehydrated in a graded series of alcohol (70%–l00%), cleared in xylene, and infiltrated in molten paraffin. Subsequently, transverse tissue sections (5–6 μm) were prepared using a rotary microtome (Leica, RM2245), stained by hematoxylin and eosin, and examined using light microscopy. The digital images were then taken by an Axio Vert A1 microscope (ZEISS).

### 2.3 EdU‐based immunohistochemistry and flow cytometry assay

5‐Ethynyl‐2′‐deoxyuridine (EdU)‐based immunohistochemistry and flow cytometry methods were performed to detect the tissue distribution characteristics and quantitative dynamic trend of cell proliferation during intestinal regeneration. EdU was injected intraperitoneally 24 h in advance at each sampling time point of intestinal regeneration. For immunohistochemistry, a dorsal incision of sea cucumber was made to expose its internal cavity and facilitates the separation of the mesentery from the body wall and intestine. In the case of non‐eviscerated sea cucumbers, the mesentery attached to the intestine and body wall was stripped away using small surgical scissors. The full length of mesentery from the anterior end (next to the esophagus) to the posterior end (next to the cloaca) of sea cucumbers were sampled and embedded in the frozen section compound (Surgipath FSC22, Leica) and stored at −80°C. Then, continuous 6‐μm thick frozen sections of these tissues were performed using a Leica CM1900 cryostat at −20°Cand immersed with pre‐cooled (4°C) acetone for 15 min. Following washed in PBST (PBS containing 0.5% Tween 20) for 10 min, the sections were permeabilized with a harsh detergent like Triton X‐100 (0.5 and 0.05%) for 30 min. After being washed again, the sections were thereafter incubated with EdU click reaction solution for 30 min at room temperature in dark. Finally, the sections were stained with Hoechst 33342 to visualize the nucleus for 10 min at room temperature and observed by a fluorescence microscope (ZEISS). The size of the regenerative tissues was calculated by the ImageJ software (http://rsbweb.nih.gov/ij/). At least three non‐consecutive tissue sections were evaluated to obtain the average of intestine rudiment area per animal. For flow cytometry assay, the single‐cell suspensions of each regenerated stage's tissues were harvested by digestion with collagenase type iv and trypsin (Gibco). These cell suspensions were then pelleted by centrifugation for 5 min at 800*g* and resuspended in 4% formaldehyde for 10 min. After three washes with PBST, these cells were incubated with EdU click reaction solution (Beyotime) for 30 min at room temperature in dark. After the last wash in a triple, the percentage of proliferation activity of these cells (10^5^ cells) was measured by a flow cytometer (Miltenyi Biotec). The data were analyzed using FlowJo software v10.6.1 (BD Biosciences).

### 2.4 Transcriptomic analysis of intestinal regeneration in *A. japonicus*

The regeneration tissues from nine individuals were respectively sampled at each sampling time point as described above and combined as one specimen for transcription sequencing. Total mRNA was performed following the protocol described by Sun et al.^19^ RNA integrity was assessed using the RNA Nano 6000 Assay Kit (Agilent Technologies). All RNA‐seq libraries were constructed using the NEB Next Ultra RNA Library Prep Kit following the manufacturer's instructions and then were subjected to paired‐end 150 bp sequencing on an Illumina Novaseq platform. Raw reads were aligned to the *A. japonicus* genome (MRZV00000000.1) using HISAT2. Potential novel transcripts were evaluated by StringTie and CPC2 v1.0.1.^20^ Expression levels of genes were calculated using Bowtie2 v2.3.4.1^21^ and RSEM v1.2.12.^22^ Differentially expressed genes (DEGs) in intestine regeneration were identified using DEseq2 v1.14.1 (*p* < 0.05 and FoldChange >2).^23^ Finally, the DEGs in different groups were analyzed by Venn diagram, cluster Profiler (3.4.4), and GO enrichment analysis.

### 2.5 Recombinant expression, antibody preparation, and western blotting

Fibroblast growth factor 4 sequences of *A. japonicus* (AjFGF4, 28–352 aa) were inserted into the expression vector pGEX‐4 T‐2 (Novagen) and then were transformed and induced in *Escherichia coli* Rosetta (DE3) by 0.5 mM isopropyl‐β‐D‐thiogalactopyranoside at 18°C overnight. The gene‐specific primers were listed in Table 1. The GST‐tag‐labeled recombinant AjFGF4 protein (rAjFGF4) was purified with GST‐Seflinose resin following the manufacturer's recommendations (Sangon Biotechnology) and confirmed by 12% SDS‐PAGE. Notably, before the final elution of proteins, we washed the column with 10 column volumes of 0.1% Triton X‐114 (Sangon Biotech) to remove most of the endotoxin contamination. Afterward, the N‐terminal GST‐tag derived from rAjFGF4 was further removed by thrombin (Solarbio) and the rabbit antiserum specific for AjFGF4 was prepared following our previously reported method.^24^ For western blotting, the protein concentrations of regenerative tissue were determined with a BCA Protein Assay Kit (Sangon). Approximately, 50 μg of protein was separated with 12% SDS‐polyacrylamide gels and transferred to 0.45‐μm ECL membrane. After blocking with 5% skimmed milk in TBST (25 mM Tris–HCl, 150 mM NaCl, and 0.1% Tween‐20) at room temperature for 2 h, the membrane was incubated with primary antibody overnight at 4°C. Then, the membrane was incubated with the corresponding secondary antibody for 1.5 h at room temperature. The membrane was stained with western Lightning‐ECL substrate (Perkin Elmer) and examined using an Image Quant LAS 4000 system (GE Healthcare Europe GmbH).

**TABLE 1:** Primer used in this study

Primer	Sequence (5′–3′)	Application
AjFGF4‐GST	F: CGGATCTGGTTCCGCGTGGATCCTCTTGTTTTCCCTCTAGTGAA	Recombinant protein
	R: GTCAGTCAGTCACGATGCGGCCGTTAGCAGACAATCTCCTCTGG	
AjFGFR2‐GST	F: CGGATCTGGTTCCGCGTGGATCCAAGACGGAGGGGACGGTTG	Recombinant protein
	R: CAGTCAGTCACGATGCGGCCGTTACCCTTGTGAGTTGTTGGCGA	
siAjFGF4‐1	GUGCUACAUUAUUGGUUAATT	RNAi
	UUAACCAAUAAUGUAGCACTT	
siAjFGF4‐2	CAGCUCGAGAACUCGAUAUTT	RNAi
	AUAUCGAGUUCUCGAGCUGTT	
siAjFGFR2‐1	GGCGAUCACUUUGUGAUAUTT	RNAi
	AUAUCACAAAGUGAUCGCCTT	
siAjFGFR2‐2	GGCGAUCACUUUGUGAUAUTT	RNAi
	AUAUCACAAAGUGAUCGCCTT	
siRNA (NC)	UUCUCCGAACGUGUCACGUTT	RNAi
	ACGUGACACGUUCGGAGAATT	
AjFGF4‐FLAG	F: CCGGACTCTAGAAAGCTTATGTTGAACACTCGGTTGG	Recombinant plasmid
	R: CTCGAGGGATCCGCTAGCCTCCTCTGGTGGATATGCA	
AjFGFR2‐EGFP	F: TCTCGGCATGGACGAGCTGTACAAGAAGACGGAGGGGACGGTTG	Recombinant plasmid
	R: TTTAAACGGGCCCTCTAGATTACCCTTGTGAGTTGTTGGCGA	
AjFGFR2‐FLAG	F: GCCTCCGGACTCTAGAAAGCTTATGAAGACGGAGGGGACGGTTG	Recombinant plasmid
	R: GTCATGGTCTTTGTAGTCCTCGAGCCCTTGTGAGTTGTTGGCGA	
qAjFGF4	F: CCTGTGTTAGTGGTCATCA	Real‐time PCR
	R: CGTCTCTATCTGGGTCTTT	
qAjBMP2	F: TCCGTCAGTGATTTTCGCT	Real‐time PCR
	R: CATTTTTCCTCTTGGTTCC	
β‐Tublin	F: GCACATCAAGCCGTCAAACTCAC	Real‐time PCR
	R: TATGCCCGCATAGCAAACATACC	

The antibodies used in this study were all listed in Table 2, including rabbit anti‐AjFGF4 serum, anti‐ERK1/2 (AF1051; Beyotime), anti‐p‐ERK (AF1891; Beyotime), anti‐JNK (66,210; Proteintech), anti‐p‐JNK (80,024; Proteintech), anti‐p38 serum, anti‐p‐p38 (AB‐21027; Elabscience Biotechnology), anti‐FGFR2 (Genscript), anti‐CDK2 (P104599; KleanAB), anti‐Cyclin A (AF6624; Beyotime), anti‐Cyclin B (GenScript), anti‐GFP (AG281; Beyotime), anti‐FLAG (M20008; Abmart), anti‐GST (AF0174; Beyotime), anti‐β‐Tubulin (M20005S; Abmart), HRP‐conjugated goat anti‐rabbit IgG (D110058; BBI), HRP‐conjugated goat anti‐mouse IgG (D110087; BBI).

**TABLE 2:** Antibodies information in this study

Antibodies	Isotype	Use	Product no.	Source
ERK1/2 rabbit monoclonal antibody	Rabbit	WB:(1:3000)	AF1051	Beyotime
Phospho‐ERK1(Thr202/Tyr204/185/Tyr187) monoclonal antibody	Rabbit	WB:(1:3000)	AF1891	Beyotime
JNK monoclonal antibody	Rabbit	WB:(1:1000)	66,210	Proteintech
Phospho‐JNK (Tyr185) antibody	Rabbit	WB:(1:500)	80,024	Proteintech
Phospho‐p38 (Thr180/Tyr182) polyclonal antibody	Rabbit	WB:(1:1000)	AB‐21027	Elabscience
Cyclin A2 rabbit polyclonal antibody	Rabbit	WB:(1:1000)	AF6624	Beyotime
CDK2 polyclonal antibody	Rabbit	WB:(1:500)	P104599	KleanAB
Anti‐β‐tubulin monoclonal antibody	Mouse	WB:(1:5000)	M20005S	Abmart
Anti‐GST mouse polyclonal antibody	Mouse	WB:(1:3000)	AF0174	Beyotime
Anti‐FLAG antibody	Rabbit	WB:(1:5000)	M20008	Abmart
GFP antibody	Mouse	WB:(1:1000)	AG281	Beyotime
HRP‐conjugated goat anti‐rabbit IgG	Rabbit	WB:(1:5000)	D110058	BBI
HRP‐conjugated goat anti‐mouse IgG	Mouse	WB:(1:5000)	D110087	BBI
P38 polyclonal antibody	Mouse	WB:(1:500)		Antiserum
AjFGF4 polyclonal antibody	Rabbit	WB:(1:500)		Antiserum
AjFGFR2 polyclonal antibody	Mouse	WB:(1:500)		Genscript
Cyclin B polyclonal antibody	Rabbit	WB:(1:1000)		GenScript

### 2.6 Plasmid construction and coimmunoprecipitation assay

The full‐length open reading frame of FGF4 (1–352 aa) and the IG2 and IG3 domain regions (81–431 aa) of FGFR2 were respectively cloned and inserted into the pcDNA3.1‐EGFP and pcDNA3.1–3 × flag expression vector (Invitrogen) to construct the following plasmids: pcDNA‐flag‐FGF4, pcDNA‐flag‐FGFR2, and pcDNA‐EGFP‐FGFR2. The primers used to amplify the gene fragments were listed in Table 1. For the coimmunoprecipitation (Co‐IP) assays, HEK‐293T cells were seeded in a T25 flask, incubated overnight, and then transfected with the indicated expression plasmids (pcDNA‐flag‐FGF4 + pcDNA‐EGFP‐FGFR2, pcDNA‐flag‐FGF4 + pcDNA‐EGFP, pcDNA‐EGFP‐FGFR2 + pcDNA‐flag) using Lipo8000 transfection reagent (C0533FT, Beyotime). At 48 h post‐transfection, the cells were washed with precooled PBS and lysed with IP cell lysis buffer containing protease inhibitor PMSF (P0013, Beyotime). After centrifugation at 13,000*g* for 20 min at 4°C, the supernatants of the cell lysates were immunoprecipitated using Protein A/G magnetic beads (P2055, Beyotime) which were bound with anti‐flag (M20008, Abmart) or anti‐GFP antibodies (AG281, Beyotime) and rotated slowly at 4°C overnight. The precipitates were washed 10 times with lysis buffer and then eluted by boiling the pellets with 5 × SDS PAGE loading buffer. Finally, the eluted samples were analyzed by immunoblotting with the indicated antibodies.

### 2.7 GST pull‐down assay

To further explore the direct combination relationship of AjFGF4 and AjFGFR2, the pull‐down assay was performed as previously reported with minor modifications.^25^ The *E. coli* Rosseta cells harboring induced expression of GST‐tag labeled AjFGF4 or GST‐tag were pelleted by centrifugation and lysed in lysis buffer via ultrasonication. After centrifugation at 13,000*g* at 4°C for 15 min, the GST‐tag and GST‐tag‐labeled AjFGF4 protein was purified by anti‐GST magnetic beads (P2138, Beyotime) and used for pull‐down assays. Subsequently, the GST magnetic beads were washed five times with lysis buffer to remove unbound proteins and were then used to incubate with HEK‐293T cell lysate overnight which was transfected with pcDNA‐flag‐FGFR2 plasmids. Finally, the beads were washed five times with 10 resin‐bed volumes of RIPA buffer for 10 min and then analyzed via immunoblot analysis to detect the flag‐FGFR2 proteins using an anti‐flag antibody (M20008, Abmart).

### 2.8 Immunofluorescence analysis

The immunofluorescence analysis was performed to detect the subcellular localization of AjFGF4 and AjFGFR2 in sea cucumber coelomocytes. The single‐cell suspensions of regenerating tissues at 12 dpe were prepared by digestion with collagenase type iv and trypsin (Gibico) for 1 h at 4°C. Cells were divided into four groups (10^6^ cells in each group) and fixed in 4% paraformaldehyde for 15 min. After washing three times with PBS, the fixed cells were then pre‐incubated with 0.5% Triton‐X‐100 in PBS for 10 min, then blocked with 5% bovine serum albumin (BSA) in PBS for 90 min at room temperature. Next, the four groups of cells were respectively incubated with the pre‐immune mouse sera (1:200 dilution), and the pre‐immune rabbit sera (1:200 dilution), the mouse anti‐FGFR2 antibody (1:200 dilution) and the pre‐immune rabbit sera (1:200 dilution), the rabbit anti‐FGF4 antibody (1:200 dilution), and the pre‐immune mouse sera (1:200 dilution), and the mouse anti‐FGFR2 antibody (1:200 dilution) and rabbit anti‐FGF4 antibody (1:200 dilution) as the primary antibodies in a moisture chamber at 37°C for 90 min. After washing three times with PBST, the cells were first incubated with FITC‐conjugated goat anti‐mouse IgG (1:1000 dilution, A0568, Beyotime) as the secondary antibodies at 37°C for 90 min. After another three washes with PBST, Cy3‐conjugated goat anti‐rabbit IgG (H1L) (1:1000 in PBS, A0516, Beyotime) was then incubated with the cells at 37°C for 90 min. Then, the nuclei were stained in DAPI (diluted to10 mg/ml in PBS, C1002, Beyotime) for 10 min at room temperature. Images were captured using a laser scanning spectral confocal microscope (ZEISS).

### 2.9 Quantitative real‐time PCR analysis

The expression profiles of AjFGF4 and AjBMP2 were detected by RT‐qPCR on an Applied Biosystem 7500 real‐time PCR system with specific primers (shown in Table 1). The cDNA concentrations of each sample were diluted 10‐fold as template for RT‐qPCR. Amplification was conducted in a 20 μl reaction volume containing 10 μl of SYBR Green I Master, 2 μl of diluted cDNA, 0.4 μl each of forward and reverse primers, 0.4 μl of ROX (Takara) and 6.8 μl of RNase‐free water to a total volume of 20 μl. The reaction mixtures were incubated at 95°C for 30 s, followed by 45 cycles of denaturation at 95°C for 5 s and extension at 60°C for 30 s. Each PCR trial was performed in triplicate with β‐tublin gene as the endogenous control. All data were analyzed relative to the β‐tublin gene by the 2^−ΔΔC*t*^ method.

### 2.10 RNA interference (RNAi) and inhibitor experiments

The specific interference oligonucleotides targeting AjFGF4 (siAjFGF4‐1 and siAjFGF4‐2), AjFGFR2 (siAjFGFR2‐1 and siAjFGFR2‐2), and its negative control siRNA (siNC) were designed and synthesized by the GenePharma Company (Table 1). For the in vivo assay, 80 μl of PBS was mixed with 10 μl of siAjFGF4 (20 μM), siAjFGFR2 (20 μM), or siNC (20 μM), and 10 μl of Lipo6000 transfection reagent (Invitrogen) as the transfection solution. Each group was set up with three replicates and each with six sea cucumbers. The mixed solutions were repeatedly injected into sea cucumber individuals six times every 2 days during intestinal regeneration. The first time of the injection of the solutions at 6 h post‐evisceration and the final injection at 10 dpe. Meanwhile, the regenerating tissues were harvested at the stage of 2, 7, and 12 dpe. The protein expression levels of AjFGF4 and AjFGFR2 were detected by western blotting and the proliferation effects of the newly formed intestinal rudiment were detected by an EdU‐based immunohistochemistry and flow cytometry as described above. For the in vitro analysis, the single‐cell suspensions of the mesentery and regenerating primordium at 12 dpe were prepared according to section immunofluorescence analysis described above. Then, these single cells were centrifuged at 800*g* and 4°C for 8 min. After being washed three times with isotonic buffer (0.001 M EGTA, 0.01 M Tri‐HCl, and 0.53 M NaCl, pH 7.6), cells were resuspended in Leiboviz's L‐15 cell culture medium (Invitrogen) containing penicillin (100 U/ml) and streptomycin sulfate (100 mg/ml). Next, for RNA interference, siAjFGF4, siAjFGFR2, or siRNA‐NC transfected with Lipofectamine 6000 (Beyotime) according to the manufacturer's recommended conditions. At 48 and 72 h post‐interference, the cells were collected and used to analyze the protein and phosphorylation levels of p38, JNK, and ERK.

We also performed inhibition assays using inhibitors. FR180204 (SD5978, Beyotime) is a type of ERK inhibitor, which inhibited the kinase activity of ERK1 and ERK2, with Ki values of 0.31 and 0.14 μM, respectively.^26^ To clarify the mechanism of intestinal regeneration, FR180204 was used to explore whether intestinal regeneration is mediated by the FGF4/FGFR2–ERK signaling pathway. The intestinal primordial cells at the stage of 12 dpe were sampled and cultured in vitro, and incubated with FR180204 or DMSO with a final concentration of 5 μM for 48 and 72 h at 18°C. Subsequently, the treated cells were collected for detecting protein expression and cell proliferation as described above.

### 2.11 AjFGF4 treatment

The purified and refolded rAjFGF4 were prepared as described above. For verifying whether AjFGF4 mediates the intestinal regeneration in *A. japonicus* through AjFGFR2, the eviscerated‐sea cucumbers were transfected with siAjFGFR2 for six times in every 2 days and supplemented it with 5 μg rAjFGF4 or BSA at 24 h post‐siAjFGFR2 transfection, and the cell proliferation levels and the size of regenerative tissues were detected. For exploring whether AjFGF4/AjFGFR2‐mediated cell proliferation through the ERK–MAPK pathway during intestinal regeneration, the single‐cell suspension of the mesentery at the stage of 12 dpe was cultured, treated with specific inhibitor FR180204 for ERK and its control DMSO for 24 h, and further supplemented it with 500 ng rAjFGF4 to detect the cell proliferation levels by the EdU staining assay. To address whether the cell proliferation could be regulated by the AjFGF4/AjFGFR2‐ERK axis through the expression of cell cycle‐related proteins, the eviscerated‐sea cucumbers were transfected with FR180204 for six times in every 2 days and supplemented with the 5 μg rAjFGF4 or BSA at 24 h post‐siAjFGFR2 transfection, and the cell proliferation levels and the protein expression level of CDK2, Cyclin A, and Cyclin B were detected.

### 2.12 Statistical analysis

All statistical analyses were performed using GraphPad Prism program (GraphPad Software, Version 9). One‐way analysis of variance (ANOVA) was applied to discern significant differences between the control and experimental groups for the percentage of EDU‐positive cell population. These results were representative of at least three independent experiments and were presented as mean ± standard deviation (SD) and significance levels were defined as *p < 0.05, **p < 0.01, ***p < 0.001, ****p < 0.0001.

## 3 RESULTS

### 3.1 Mesentery is the center of intestinal regeneration in *A. japonicus* by morphological observation

**FIGURE 1:**
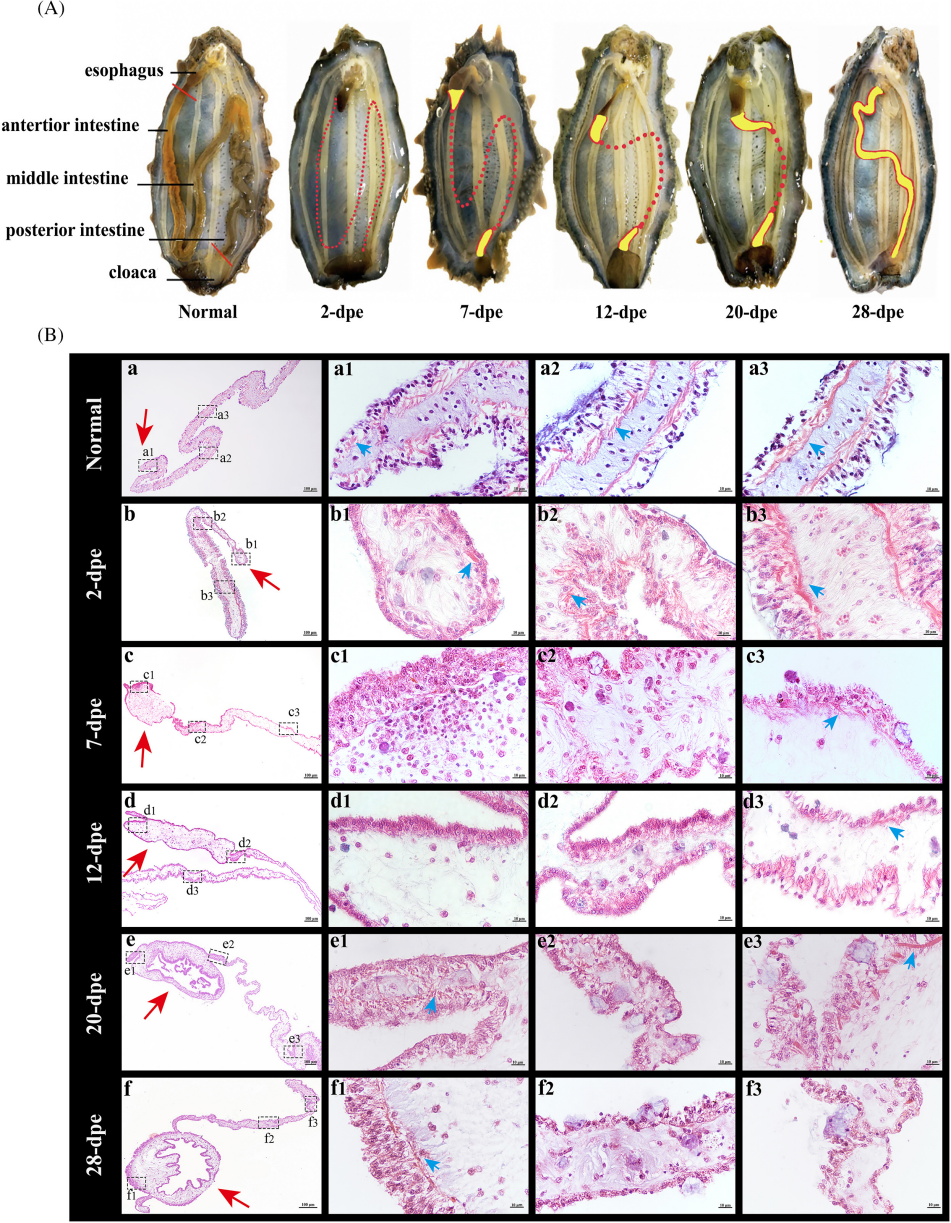
Histological observation reveals the intestinal regeneration process of *Apostichopus japonicus*. (A) Schematic diagram of the internal anatomy of *A. japonicus* in normal and in different regenerative stages (from 2 to 28 dpe). Sea cucumbers were dissected along the body length, at the right side of the mid‐dorsal, and flattened. Red solid line: the intestine breaks at its anterior end from the esophagus and at the posterior end from the cloaca. Red broken line: the edge of the mesentery; yellow area: newly formed intestinal lumen; (B) transverse tissue sections of the mesentery and regenerating intestine at normal, 2, 7, 12, 20, and 28 dpe were stained with hematoxylin–eosin (a–f). The free edge of the mesentery is marked with a red arrow. At 2 dpe, the epithelium covers the tip of the mesentery and the tear edge of the mesentery gradually recovered (b). At 7 dpe, a small enlargement can be observed at the mesenterial tip (c). At 12 dpe, the intestinal rudiment has increased considerably in size (d). At 20 dpe, the lumen has formed, but the epithelial fold has not been fully formed (e). At 28 dpe, the intestinal rudiment continued to grow in size and all tissue layers of the mature intestine can be found within the rudiment (f). a_1–3_–f_1–3_ are the high magnification images of the black boxes in a–f. The muscular layer is marked by a blue arrow. Bar (a–f) = 100 μm, bar (a_1–3_–f_1–3_) = 10 μm

To investigate the intestine regeneration process of *A. japonicus* following spontaneous evisceration, the morphological changes of internal organs during the period of self‐evisceration and regeneration were observed for 28 days. Our result showed that only a part of the esophagus, cloaca, and a continuous mesentery remained in the body cavity left after evisceration (Figure 1A). Notably, we found that a new lumen was finally generated at the free end of the mesentery, which indicated the key roles of mesentery during intestinal regeneration. To further explore the connection between the newly formed intestine and the mesentery, we performed the tissues paraffin sections and HE staining to describe the basic histomorphology changes throughout the process of intestinal regeneration. Figure 1B, a–f illustrated the overall histomorphology changes of the mesentery in normal and in different regenerative stages (from 2 to 28 dpe), and Figure 1B, a_1–3_–f_1–3_ was the high magnification images of the black boxes in Figure 1B, a–f. As shown in Figure 1B, compared to the normal mesentery (Figure 1B, a), regeneration began with the healing of the far edges of the mesentery within the first 2 dpe. During this period, with the migration of epithelial cells, some of the epithelial cells began to ingress into the connective tissue at the tip of the mesentery and the cut edge of the mesentery gradually recovered (Figure 1B, b_1_). More interesting was that the muscle layer disappeared from the growth along the mesentery up to its tip (Figure 1B, b_1_, blue arrowhead), which might involve a fate transition of cell dedifferentiation. Subsequently, the first indication of swelling of the distal mesentery was observed at the stage of 7 dpe (Figure 1B, c), and ingesting cells from the epithelial layer of the edge of the mesentery formed a mass of cells adjacent to the tip of the mesenterial thickening (Figure 1B, c_1_). The muscle layer gradually disappeared from the free end of the mesentery, and only a little was observed near the end of the body cavity wall (Figure 1B, c_1–3_, blue arrowhead). In the following days (12 dpe), there was significant growth in the size of the thickening that would form the intestinal rudiment. This rudiment acquired an elongated oval morphology composed of a large number of cells within an inner connective tissue and starts, although in some cases or sections the growth could be somewhat irregular (Figure 1B, d). At the stage of 20 dpe, the lumen had already formed (Figure 1B, e), and the mucosal epithelium had regenerated by the invasion of tubular outgrowths of mucosal epithelium from the esophagus and the cloaca. Although the epithelial fold had not been fully formed, all tissue layers of the mature intestine particularly the muscle layer could be observed within the rudiment (Figure 1B, e_1_). In the late stage of regeneration (28 dpe), the intestinal rudiment continued to grow and acquired a digestive tract shape (Figure 1B, f). The intestinal folds were gradually increased and the basic structural format of the intestine (from the esophagus to cloaca) had already been established (Figure 1B, f_1_). As noted above, our results confirmed that the mesentery played an important role in the process of intestinal regeneration and the new intestinal rudiment was a swelling developed at the free edge of the mesentery in *A. japonicus*, which might involve a series of cellular events (cell dedifferentiation, cell migration, cell proliferation, cell differentiation).

### 3.2 Intestinal regeneration involves in massive cell proliferation

**FIGURE 2:**
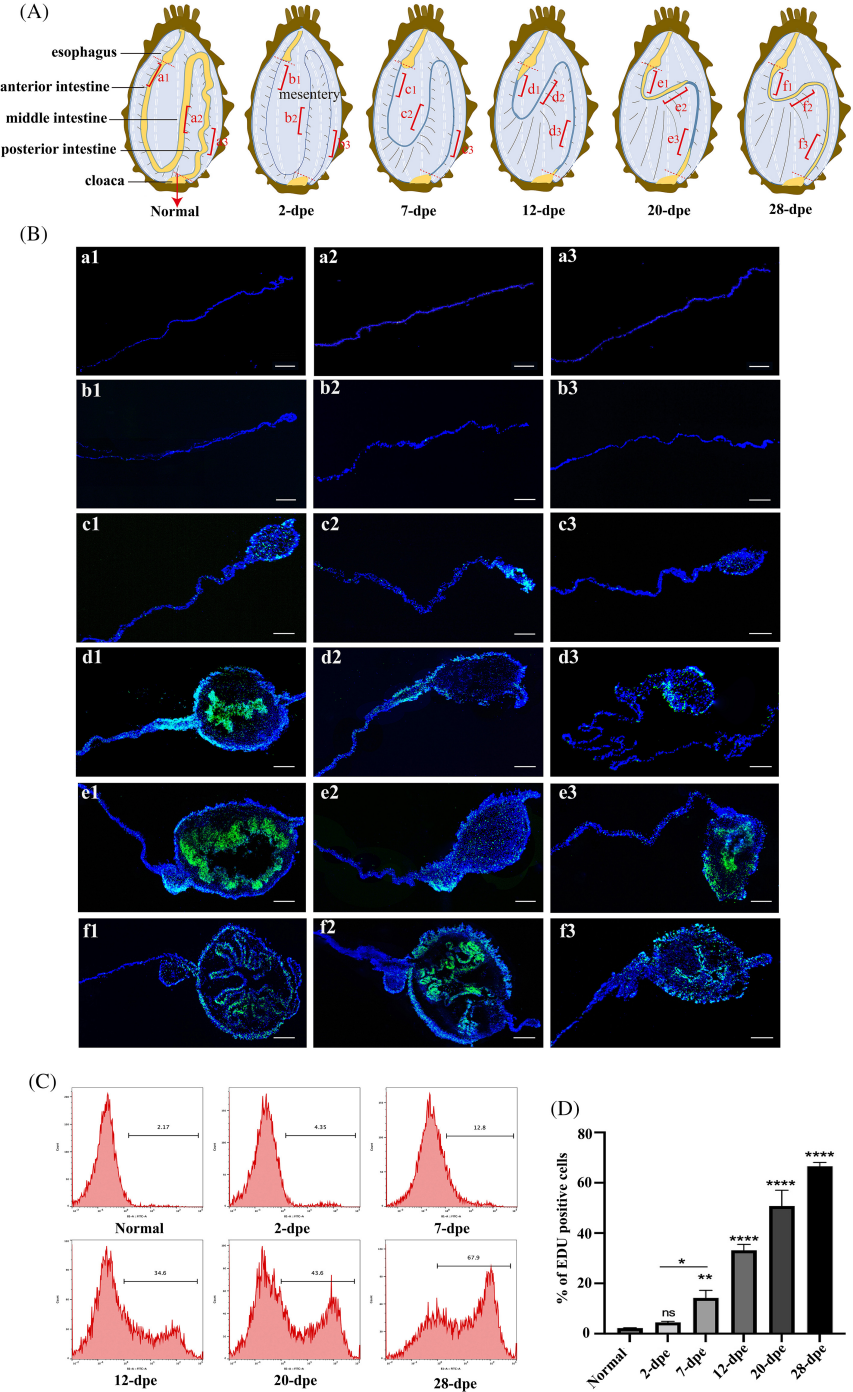
Cell proliferation in *Apostichopus japonicus* during intestinal regeneration. (A) Schematic reconstruction of the sequence of events during regeneration of the intestinal system in *A. japonicus*. The diagram presents only the organs involved in this process. Other organs, such as the right respiratory tree, have been removed. a_1–3_–f_1–3_ marked in the schematic correspond to the sampling sites in (B), respectively. (B) EdU assay was performed to detect the cell proliferation levels in normal and different regenerative stages (2–28 dpe). The sampling locations of dorsal, ventral, and lateral mesentery at each stage of intestinal regeneration were stained. Blue color indicates Hoechst‐stained nuclei, and green indicates EdU‐stained proliferating cells. Bar = 200 μm. (C) EdU‐based flow cytometry was used to analysis the changes in cell proliferation levels of normal and different regenerative stages (2–28 dpe). (D) The number of EdU‐labeled cells in the normal group and five regeneration groups were comparatively analyzed and represented by a histogram. Results are the mean ± SD from three independent experiments. In each experiment, the cells of three *A. japonicus* were analyzed. Per sample, 10,000 events were acquired on an FACS flow cytometer. “ns” indicates no significant difference versus the control. *p < 0.05; **p < 0.01; ****p < 0.0001 versus the control

According to our histochemical results described in Figure 1B, the swelling of the end of the mesentery was necessary for the process of the intestine regeneration and required a mass number of cells formed under the control of cell proliferation. Thus, we further explored whether cell proliferation was involved in intestinal regeneration and contributed to the thickening of the mesenterial tip and the formation of the intestinal rudiment. The dorsal, ventral, and lateral mesentery at each stage of intestinal regeneration (marked in red, a_1–3_–f_1–3_) were sampled as shown in Figure 2A. The cell proliferation was assayed by an EdU method (Figure 2B) and the size of the regenerative tissues was shown in Figure S1. In the early stage of the intestine regeneration, even though the cut reparation had begun, hardly any proliferation could be found in the remaining mesenteries at 2 dpe (Figure 2B, b_1_–b_3_). Then, at the stage of 7 dpe, the number of EdU‐labeled cells increased in all areas of the intestinal rudiment, especially at the free end of the mesentery, which correlated with an increase in its size (Figure 2B, c_1_–c_3_). From the 12 dpe, the cell masses were observed in all samples in the form of thickenings at the free end of the mesentery. At this time, a very narrow cavity was found in the anterior regenerated primordium, which was near the end of the esophagus. The distribution of EdU‐labeled dividing cells was somewhat heterogeneous with more labeled cells found in the mesothelial layer and luminal epithelium of the distal mesenterial area. However, during this period, the regeneration primordia of the medial and posterior sides were smaller than that of the anterior side, and no cavity formation was observed. The proliferating cells of the mesentery were distributed in the mesothelial layer at the end of the mesentery (Figure 2B, d_1_–d_3_). At the stage of 20 dpe, except for the regenerated primordia in the middle, cavities were formed in both the front and rear ends, and proliferating cells exploded in large numbers, and were distributed in the cavities of the terminal enlargement, which seemed to prepare for the formation of intestinal epithelial cells (Figure 2B, e_1_–e_3_). Afterward, in the later stages of intestinal regeneration (28 dpe), the entire intestinal rudiment including the anterior, middle, and posterior had formed a cavity, and EdU‐labeled dividing cells were mainly distributed in the mesothelial layer and the epithelial layer that has formed folds (Figure 2B, f_1_–f_3_).

We also performed EdU‐based flow cytometry to detect the changes in cell proliferation during intestinal regeneration under the same conditions. As shown in Figure 2C, the results of cell proliferation by the EdU‐based flow cytometry were in line with those of immunohistochemistry described above. In the early stage (2 dpe) of intestinal regeneration, the increment of cell proliferation was not statistically significant (4.48% ± 0.40%) compared with the normal group (3.46% ± 0.95%). At the stage of 7 and 12 dpe, the proliferation rate reached 14.2% ± 3.06% and 33.13% ± 2.37%, respectively. The value soared to 50.77% ± 6.26% at the stage of 20 dpe. At this time, the number of proliferating cells had exceeded half of all cells, indicating that the tissue was growing vigorously. At the stage of 28 dpe, although the intestinal tract had penetrated, the cell proliferation rate did not decrease, reaching 66.53% ± 1.58%, indicating that proliferating cells were still needed in the later stage of intestinal function formation. Taken together, our results demonstrated that intestinal regeneration was accompanied by large numbers of cell proliferation.

### 3.3 FGF4 regulates cell proliferation during intestinal regeneration

**FIGURE 3:**
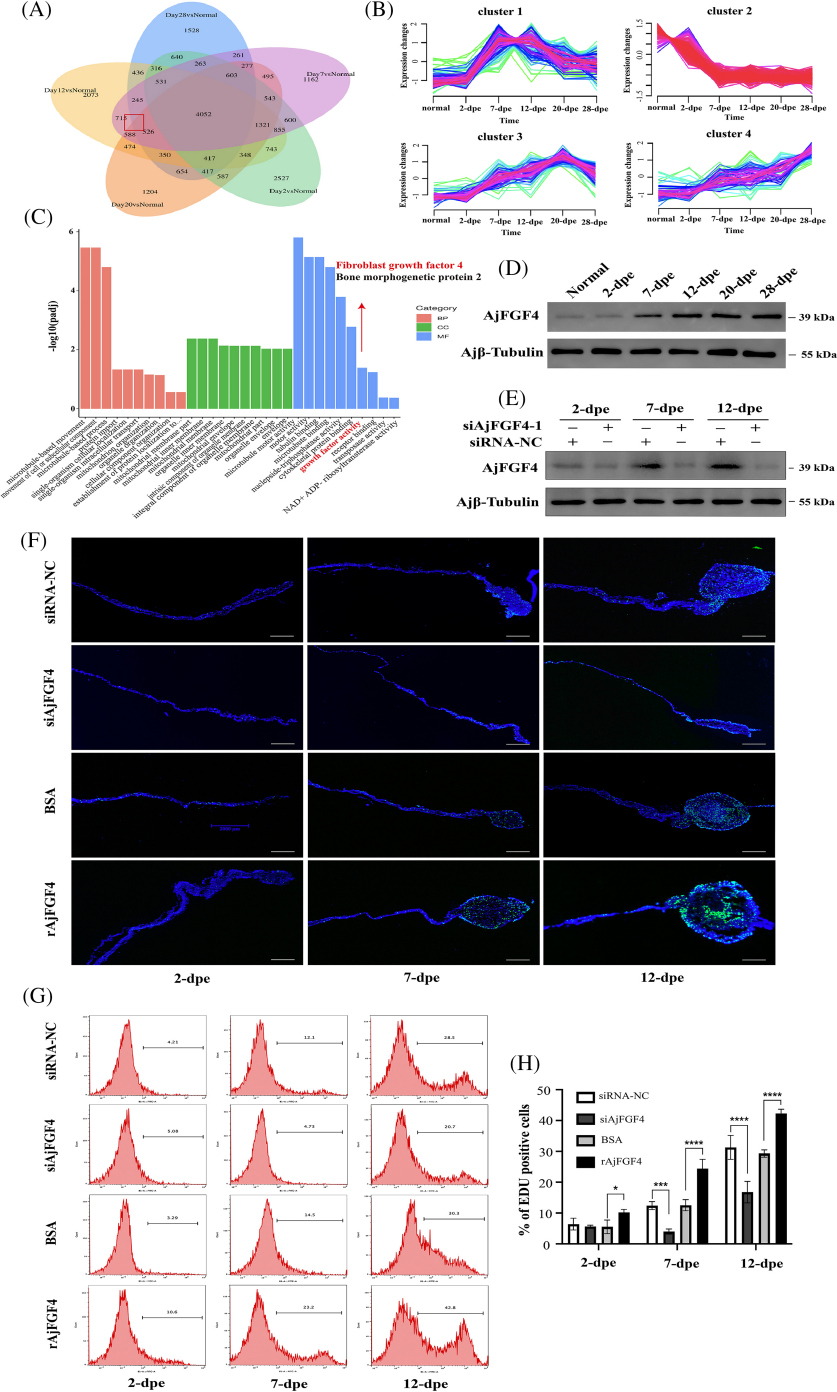
AjFGF4 is identified as a target to regulate cell proliferation during intestinal regeneration. (A) A Venn diagram showed overlapping genes between the normal group and different regenerative groups. (B) Expression changes mean normalized expression behavior of highlighted clusters from the intersection of four regeneration groups (7 dpe vs. normal, 12 dpe vs. normal, 20 dpe vs. normal, 28 dpe vs. normal). (C) Gene ontology enrichment analysis of DEGs of upregulated clusters (Clusters 1, 3, 4). Ontology domains comprise biological process (BP), cellular component (CC), and molecular function (MF). (D) The protein expression level of AjFGF4 in the regenerating mesentery and intestine in the normal and different regenerative groups. (E) Sea cucumbers were transfected with siNC or siAjFGF4 for six times in every 2 days post‐evisceration treatment. The protein levels of AjFGF4 in the regenerating mesentery and intestine at 2, 7, and 12 dpe were determined by western blotting with anti‐AjFGF4 Ab and anti‐Ajβ‐Tubulin Ab as the internal reference. (F) EdU assay detection of cell proliferation and the size of the regenerative tissues in the groups of siRNA‐NC, siAjFGF4, BSA, and rAjFGF4 groups at 2, 7, and 12 dpe. The regeneration tissues in each group were taken from the same location in the anterior end (next to the esophagus) of the sea cucumber. Blue color indicates Hoechst‐stained nuclei, and green indicates EdU‐stained proliferating cells. Bar = 200 μm. (G) Flow cytometry analysis for detecting EdU incorporation into cells. (H) Comparison of the EdU‐labeled cells in siRNA‐NC, siAjFGF4, BSA and rAjFGF4 groups at 2, 7, and 12 dpe. Results are the mean ± SD of three independent experiments. In each experiment, the cells of three *Apostichopus. japonicus* were analyzed. Per sample, 10,000 events were acquired on a FACS flow cytometer. *p < 0.05, **p < 0.01, ***p < 0.001, ****p < 0.0001

The transcriptomic data derived from the regeneration tissues from the normal group and five regeneration groups (2, 7, 12, 20, and 28 dpe) were used to investigate the underlying molecular pathways involved in cell proliferation during intestine regeneration. The raw data has been uploaded to NCBI Sequence Research Archive under accession number SAMN29388289. The result showed that 14,763, 13,037, 13,990, 12,856, and 11,516 DEGs were detected (*p* < 0.05) at the stage of 2‐, 7‐, 12‐, 20, and 28 dpe, respectively. In view of the fact that the cell proliferation of 2 dpe is not obvious in the results (Figure 2), we excluded DEGs of 2 dpe from the Venn diagram analysis and obtained 526 co‐expressed DEGs (Figure 3A). Then, cluster hierarchy was applied to analyze these 526 DEGs, and three clusters were identified with coherent upregulation of genes (Clusters 1, 3, and 4) and one coherent downregulation of genes (Cluster 2) (Figure 3B). Further GO terms enrichment analysis identified two possible candidate genes “Fibroblast growth factor 4 (FGF4)” and “Bone morphogenetic protein 2 (BMP2)” from Clusters 1, 3, and 4, which were associated with the molecular function of “growth factor activity” (Figure 3C). However, in view of the RT‐qPCR result that upregulated fold of AjFGF4 is higher than that of AjBMP2 gene, as well as the upregulated trend of AjFGF4 is consistent with that of cell proliferation (Figure S2), we first selected AjFGF4 as a target in this research. The nucleotide, amino acid sequences, and the domain analysis of AjFGF4 were shown in Figure S3. The SMART program analysis result showed that AjFGF4 contains a signal peptide and a classic FGF domain.

The protein levels of AjFGF4 in the mesentery and the newly formed intestinal rudiment during the process of the intestine regeneration were detected by WB. As shown in Figure 3D, the protein expression level of AjFGF4 was not significant at 2 dpe, but gradually increased from Day 7 to 28 dpe. To further confirm the effect of AjFGF4 on cell proliferation during intestine regeneration, a specific siRNA targeting AjFGF4 (siAjFGF4‐1 and siAjFGF4‐2) (Table 1) were synthesized and injected into *A. japonicus* for six times in every 2 days post‐self‐evisceration treatment. An equal amount of siNC was injected as a control. As shown in Figures 3E and S4, the protein levels of AjFGF4 in the mesentery and the newly formed intestinal rudiment were much increased at the stage of 7 and 12 dpe during intestinal regeneration in siNC group, while its expression was significantly reduced in siAjFGF4 treatment group. In the case of demonstrating that there is no off‐target effect, we selected siAjFGF4‐1 to further investigated whether AjFGF4 mediates intestinal regeneration by an EdU method. As shown in Figure 3F, EdU labeling showed that there was no major difference among the siRNA‐NC, siAjFGF4, BSA, and rAjFGF4 group at the first 2 dpe. By contrast, at the 7 and 12 dpe, the numbers of EdU‐labeled cells increased in all areas of the intestinal rudiment and the lumen had been initially formed both in the siRNA‐NC, rAjFGF4 and BSA group, while such phenomenon was hardly observed in the siAjFGF4 group. Compared with the siRNA‐NC group, the size of the regenerated tissue was significantly inhibited in the siAjFGF4 group. Moreover, compared with the BSA group, the size of the regenerated tissue were significantly improved in the rAjFGF4 group. The size of the regenerated intestinal primordium was calculated and summarized in Figure S5, and its dynamic trend among groups was the same as that of cell proliferation. Then, the EdU‐based flow cytometry assay was also performed to confirm the roles of AjFGF4 in mediating cell proliferation rate during intestine regeneration. The results of cell proliferation trend which detected by the EdU‐based flow cytometry assay (Figure 3G) were highly correlated with the results detected by the immunofluorescence (Figure 3E). According to the statistical results (Figure 3H), besides the stage of 2 dpe, there was a significant difference in the levels of cell proliferation between the siRNA‐NC and siAjFGF4 groups. At the stage of 7 and 12 dpe, the cell proliferation levels in the siRNA‐NC group reached 12.40% ± 1.28% and 31.27% ± 3.88%, while that in the siAjFGF4 group was only 3.99% ± 0.84% and 16.77% ± 3.48%, respectively. There was also a difference in cell proliferation between BSA and rAjFGF4 groups. At the stage of 2, 7, and 12 dpe, the cell proliferation levels in the BSA group reached 5.52% ± 2.21%, 12.53% ± 1.82% and 29.30% ± 1.18%, while that in the rAjFGF4 group increased to 10.20% ± 0.96%, 24.37% ± 3.02% and 42.30% ± 1.42%. Taken together, these data highly demonstrated that AjFGF4 affected intestinal regeneration by regulating cell proliferation in *A. japonicus*.or BSA and rAjFGF4 group.

### 3.4 AjFGFR2 serves as the receptor of AjFGF4

**FIGURE 4:**
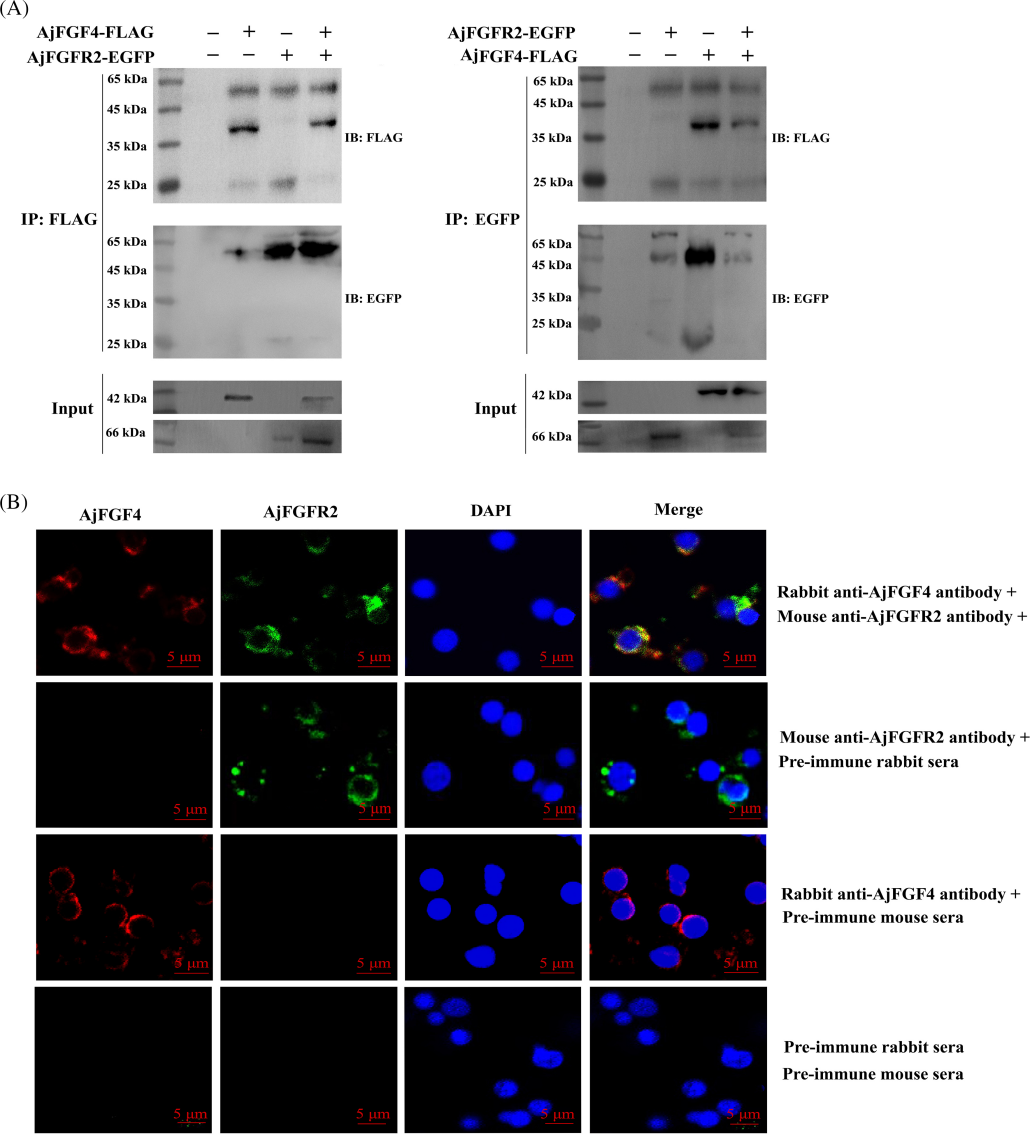
AjFGF4 interacts with AjFGFR2. (A) The binding capacity of AjFGF4 and AjFGFR2 was detected by the co‐IP assay in vitro. HEK293T cells were transfected with 5 μg of empty vector or AjFGF4‐flag and AjFGFR2‐EGFP plasmids, respectively. At 48 h posttransfection, cell lysates were immunoprecipitated with protein A/G magnetic beads which were bound with anti‐flag or anti‐GFP antibodies and immunoblotted with anti‐flag and anti‐GFP Abs by western blotting. (B) Colocalization of AjFGF4 and AjFGFR2 in single‐cell suspensions of regenerating tissues at 12 dpe was analyzed by laser confocal microscopy. Red color represents Cy3‐labeled AjFGF4, green represents FITC‐labeled AjFGFR2, and blue represents DAPI‐stained nuclei. The last row indicates the images in the first three panels as a digital overlay to visualize colocalization. Single‐cell suspensions from the first to the fourth rows were, respectively, incubated with the rabbit anti‐AjFGF4 Ab and mouse anti‐AjFGFR2 Ab, the mouse anti‐AjFGFR2 Ab and the pre‐immune rabbit sera, the rabbit anti‐AjFGF4 Ab and the pre‐immune mouse sera, and the pre‐immune rabbit sera and the pre‐immune mouse sera as primary Abs, and Cy3‐conjugated goat anti‐rabbit IgG and FITC‐conjugated goat anti‐mouse IgG were served as secondary Abs. Scale bar = 5 μm.

In higher vertebrates, FGFs function by interacting with their specific receptors (FGFRs) to activate different signals in various cells.^27^ Through the annotation analysis of *A. japonicus* genomic (Bioproject: PRJNA354676), a total of four FGFR candidates were found, including AjFGFR1 (MRZV01002165.1:51,357|60,782), AjFGFR2 (MRZV01000361.1:158,510|197,505), AjFGFR3 (MRZV01000524.1:302,688|308,899), and AjFGFR4 (MRZV01000575.1:625,607|649,597). First, the AphaFold2 was used to predict the protein structure prediction.^28^ Then, the ZDOCK server (https://zdock.umassmed.edu/) was performed to identify the possible binding partners of these four AjFGFR candidates to AjFGF4.^29^ Finally, Pymol, an open‐source molecular graphics tool was used to visualize the docking results. The 2D results of molecular docking showed that AjFGFR2 and AjFGF4 possessed the most hydrogen bonds (12 H‐bond), which was more than AjFGF4 with AjFGFR1 (4 H‐bond), AjFGFR3 (3 H‐bond), and AjFGFR4 (5 H‐bond) (Figure S6), which indicated that the binding effect of AjFGFR2 and AjFGF4 might be stronger. Moreover, the 3D model diagram of molecular docking between AjFGFR2 and AjFGF4 was shown in Figure S6B. The sequence characteristics of AjFGFR2 were shown in Figure S7. The conserved domains analysis showed that AjFGFR2 had a conserved intracellular STYKc domain, a transmembrane domain and an extracellular three Ig domains named Ig1, Ig2 and Ig3 (Figure S7). Using either a captured anti‐GFP or anti‐flag antibody, AjFGF4‐flag and AjFGFR2‐GFP were detected to be coprecipitated in the transfected HEK293T cell lysates by Co‐IP assay (Figure 4A). Subsequently, we conducted a GST‐pull down assay to further examine whether AjFGF4 interacted with AjFGFR2. To achieve this goal, we first specifically bound GST‐tag labeled AjFGF4 protein or GST‐tag protein using anti‐GST magnetic beads, and then incubated the cell lysates transfected with pcDNA‐flag‐FGFR2 plasmids. As expected, we observed a single band with the expected molecular weight using the anti‐flag antibody, while no bands were detected in the control group (Figure S8). We next explored the subcellular localization of AjFGF4 and AjFGFR2 in single‐cell suspensions of the mesentery at the stage of 12 dpe using laser confocal microscopy. As shown in Figure 4B, the colocalization of FITC‐labeled AjFGFR2 and Cy3‐labeled AjFGF4 were observed on the cytoplasm and membrane (yellow color).

### 3.5 AjFGF4 regulates *A. japonicus* cell proliferation depending on the recognition of AjFGFR2 during intestinal regeneration

**FIGURE 5:**
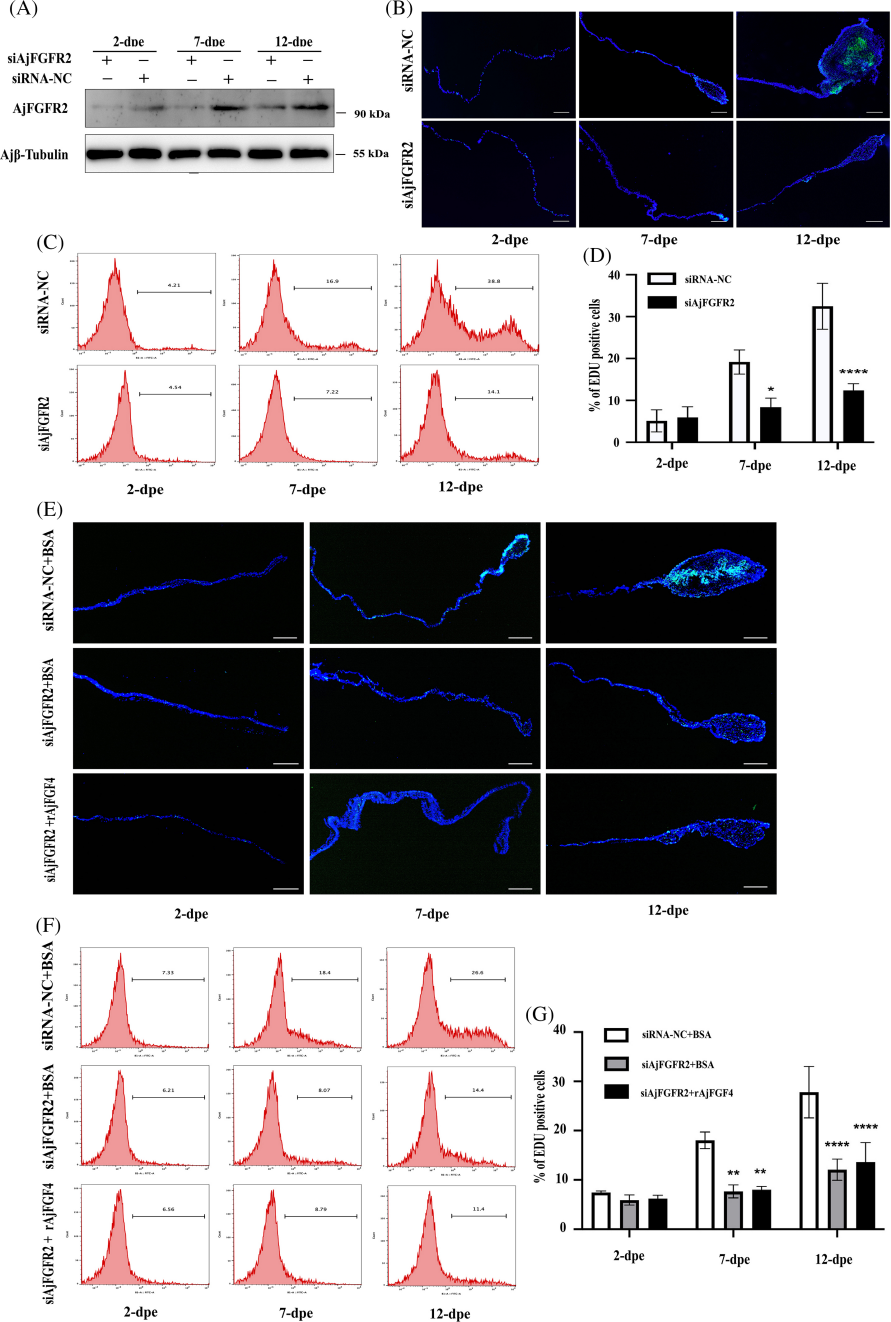
AjFGF4 regulates *Apostichopus japonicus* cell proliferation depending on the recognition of AjFGFR2 during intestinal regeneration. (A) Sea cucumbers were transfected with siRNA‐NC or siAjFGFR2 for six times in every 2 days postevisceration treatment. The protein levels of AjFGF4 in the regenerating mesentery and intestine at 2, 7, and 12 dpe were determined by western blotting with anti‐AjFGFR2 Abs and anti‐Ajβ‐Tubulin Ab as the internal reference. (B) EdU assay detection of cell proliferation and the size of the regenerative primordium in the siRNA‐NC group and siAjFGFR2 group at 2, 7, and 12 dpe. The regeneration tissues in each group were taken from the same location in the anterior end (next to the esophagus) of the sea cucumber. Blue color indicates Hoechst‐stained nuclei, and green indicates EdU‐stained proliferating cells. Bar = 200 μm. (C) Flow cytometry analysis for detecting EdU incorporation into cells at 2, 7, and 12 dpe in the siAjFGFR2 group and siRNA‐NC group. (D) Comparison of the number of EdU‐labeled cells at 2, 7, and 12 dpe between the siAjFGFR2 and siRNA‐NC groups. (E) EdU assay detection of cell proliferation and the size of regenerative tissues in the groups of siRNA‐NC + BSA, siAjFGFR2 + BSA, siAjFGFR2 + rAjFGF4 at 2, 7, and 12 dpe. Blue color indicates Hoechst‐stained nuclei, and green indicates EdU‐stained proliferating cells. Bar = 200 μm. (F) Comparison of the numbers of EdU‐labeled cells in the siRNA‐NC + BSA, siAjFGFR2 + BSA, siAjFGFR2 + rAjFGF4 group. All data are presented as the mean ± SD of three independent experiments. In each experiment, the cells of three *A. japonicus* were analyzed. Per sample, 10,000 events were acquired on an FACS flow cytometer. *p < 0.05, **p < 0.01, ***p < 0.001, ****p < 0.0001

To further explore whether AjFGF4 mediated intestinal regeneration by recognizing AjFGFR2, we investigated the changes in the levels of cell proliferation during intestinal regeneration post‐siAjFGFR2‐1/ siAjFGFR2‐2 or siRNA‐NC treatment. As shown in Figures 5A and S9, in the siRNA‐NC group, the AjFGFR2 protein levels in the tissues of the mesentery and the newly formed intestinal rudiment were significantly increased during intestinal regeneration, while this result was reduced at the stage of 7 and 12 dpe post‐siAjFGFR2 treatment. In the case of demonstrating that there is no off‐target effect, we selected siAjFGFR‐1 to further investigate whether AjFGFR2 mediated intestinal regeneration by EdU‐based immunohistochemistry assay. As shown in Figures 5B and S10, we observed that the size of the regenerative intestinal rudiment and the number of EdU‐labeled cells in the siAjFGFR2 group were much reduced at the stage of 7 and 12 dpe, compared to those in the siRNA‐NC group. It was subsequently confirmed by the EdU‐based flow cytometry assay under the same conditions. The flow cytometry analysis results indicated that downregulation of AjFGFR2 significantly decreased the cell proliferation levels at the stage of 7 and 12 dpe, relative to that in the siRNA‐NC groups (Figure 5C,D). These results demonstrated that AjFGFR2 was an important molecule in the regulation of cell proliferation during intestinal regeneration of *A. japonicus*.

To further verify that AjFGF4 mediated the intestinal regeneration in *A. japonicus* through AjFGFR2, we transfected siAjFGFR2 in eviscerated‐sea cucumbers six times every 2 days and then supplemented it with the same dose of rAjFGF4 or BSA at 24 h post‐siAjFGFR2 transfection. As shown in Figure 5B–G, both the EdU‐based flow cytometry assay and immunohistochemistry analysis results showed that compared with the control group, the proliferative cells in the tissues of the mesentery and the newly formed intestinal rudiment of siAjFGFR2‐ (Figure 5B–D) and rAjFGF4 + siAjFGFR2‐treated (Figure 5E–G) sea cucumbers were significantly inhibited. However, there was no significant difference in the cell proliferation levels between the sea cucumbers treated with siAjFGFR2 and rAjFGF4 + siAjFGFR2, indicating that AjFGF4 mediated cell proliferation during intestine regeneration via AjFGFR2. At the same time, the size of the intestine rudiment area corresponding to Figure 5E was calculated and summarized in Figure S11. The result showed that the intestine rudiment area in siAjFGFR2 + BSA group was significantly reduced compared to siRNA‐NC + BSA group, while the area in siAjFGFR2 + rAjFGF4 group was significantly decreased compared to siRNA‐NC + BSA group. In each experimental group, the size of the regenerated intestine had the same trend as the cell proliferation.

### 3.6 AjFGF4/AjFGFR2 regulates the cell proliferation through the ERK–MAPK pathway

**FIGURE 6:**
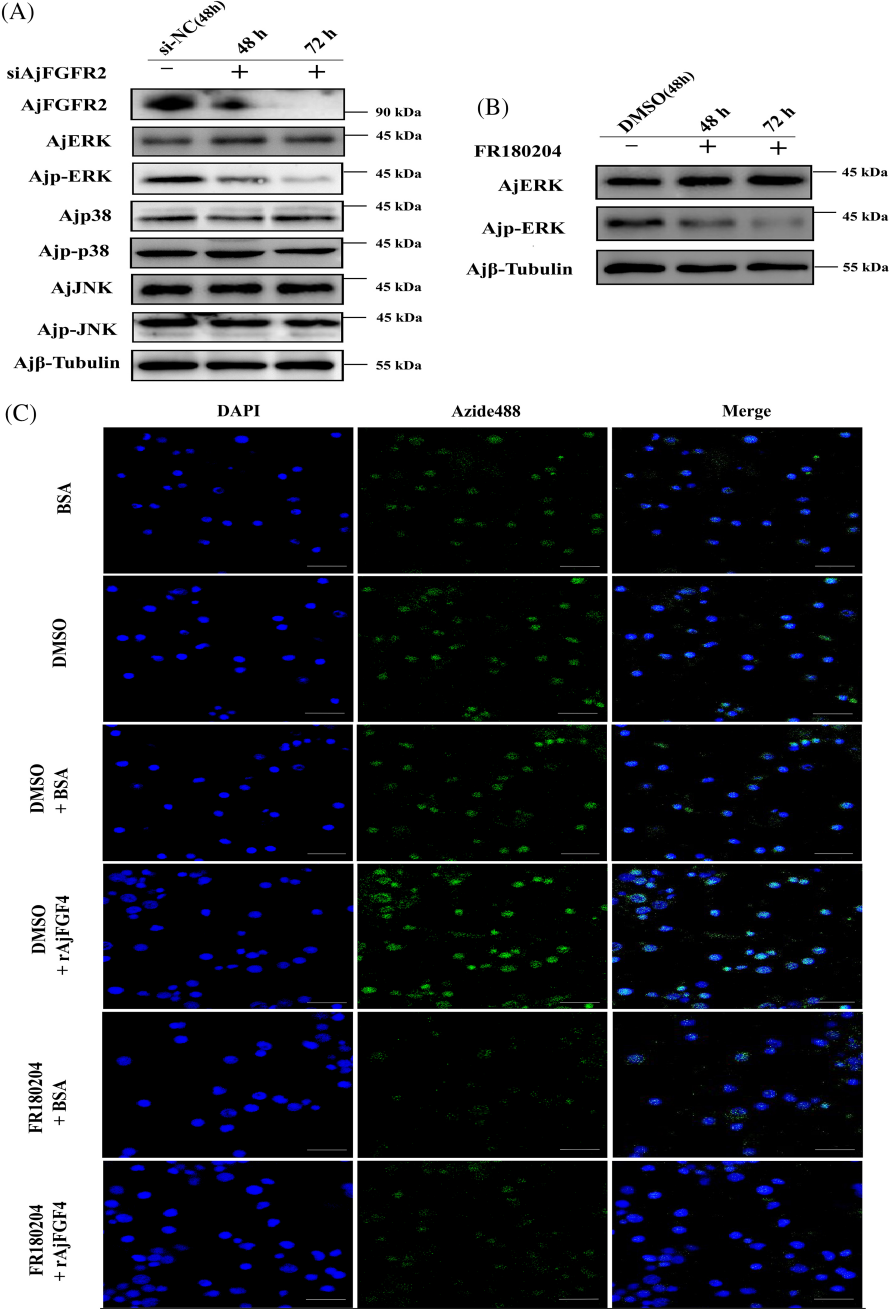
AjFGF4/AjFGFR2‐mediated cell proliferation is depending on ERK–MAPK pathway during intestinal regeneration. (A) The protein or phosphorylation level of AjFGFR2, ERK, p38, and JNK in the single‐cell suspension of the mesentery at the stage of 12 dpe were detected at 48 and 72 h post‐siAjFGFR2 treatment by western blotting using anti‐p38 Ab and anti‐p‐p38 Ab, anti‐JNK Ab and anti‐p‐JNK Ab, anti‐ERK1/2 Ab, anti‐p‐ERK1/2 Ab, and anti‐Ajβ‐Tubulin Ab as the internal reference, respectively. (B) The protein and phosphorylation levels of ERK in the single‐cell suspension of the mesentery at the stage of 12 dpe were detected at 48 and 72 h after being treated with FR180204 (ERK inhibitor, 5 μM) by western blotting using anti‐ERK1/2 Ab, anti‐p‐ERK Ab, and anti‐Ajβ‐Tubulin Ab as the internal reference. (C) EdU assay detection of cell proliferation in the mesentery single‐cell suspension at the stage of 12 dpe after being treated with BSA, DMSO, DMSO + BSA, DMSO + rAjFGF4, FR180204 + BSA, and FR180204 + rAjFGF4. Blue color indicates Hoechst‐stained nuclei, and green indicates EdU‐stained proliferating cells. The last row shows the images in the first two panels as a digital overlay to visualize colocalization by laser confocal microscopy. Scale bar = 10 μm. In each experiment, the sample of six *Apostichopus japonicus* was analyzed and each experiment was repeated three times.

The MAPK signaling pathway was reported to be closely associated with cell survival, proliferation, and differentiation during regeneration in several animals, such as hydra and zebrafish.^30^ ERK, JNK, and p38 were mitogen‐activated kinases in the signaling pathway and could be triggered by phosphorylation. To elucidate the molecular mechanism underlying AjFGF4/AjFGFR2‐mediated cell proliferation during intestinal regeneration, we cultured the single‐cell suspension of the mesentery at the stage of 12 dpe in vitro and further explored the protein and phosphorylation levels of ERK, JNK, and p38 pathways post‐siAjFGFR2 treatment. The results of immunoblot analysis showed that there was no significant difference in the protein levels of JNK, p38, and ERK, and in the phosphorylation levels of JNK and p38 in the siAjFGFR2 group and the siRNA‐NC group. However, the phosphorylation levels of ERK were significantly inhibited by treating with siAjFGFR2 under the same conditions (Figure 6A). To further confirm this result, we treated the single‐cell suspension with specific inhibitor FR180204 for ERK and its control DMSO for 24 h and further supplemented it with rAjFGF4 to detect the cell proliferation levels by the EdU staining assay. The results showed that the phosphorylation level of ERK was significantly suppressed, but not the protein levels (Figure 6B). In this condition, we detected that there was no significant difference in the cell proliferation levels between the sea cucumbers treated with FR180204 + BSA and FR180204 + rAjFGF4 (Figure 6C). Taken together, these results suggested that AjFGF4/AjFGFR2 regulated the cell proliferation through the ERK–MAPK pathway during intestinal regeneration.

### 3.7 AjFGF4/AjFGFR2‐ERK induced the expression of cell cycle‐related proteins in cell proliferation

**FIGURE 7:**
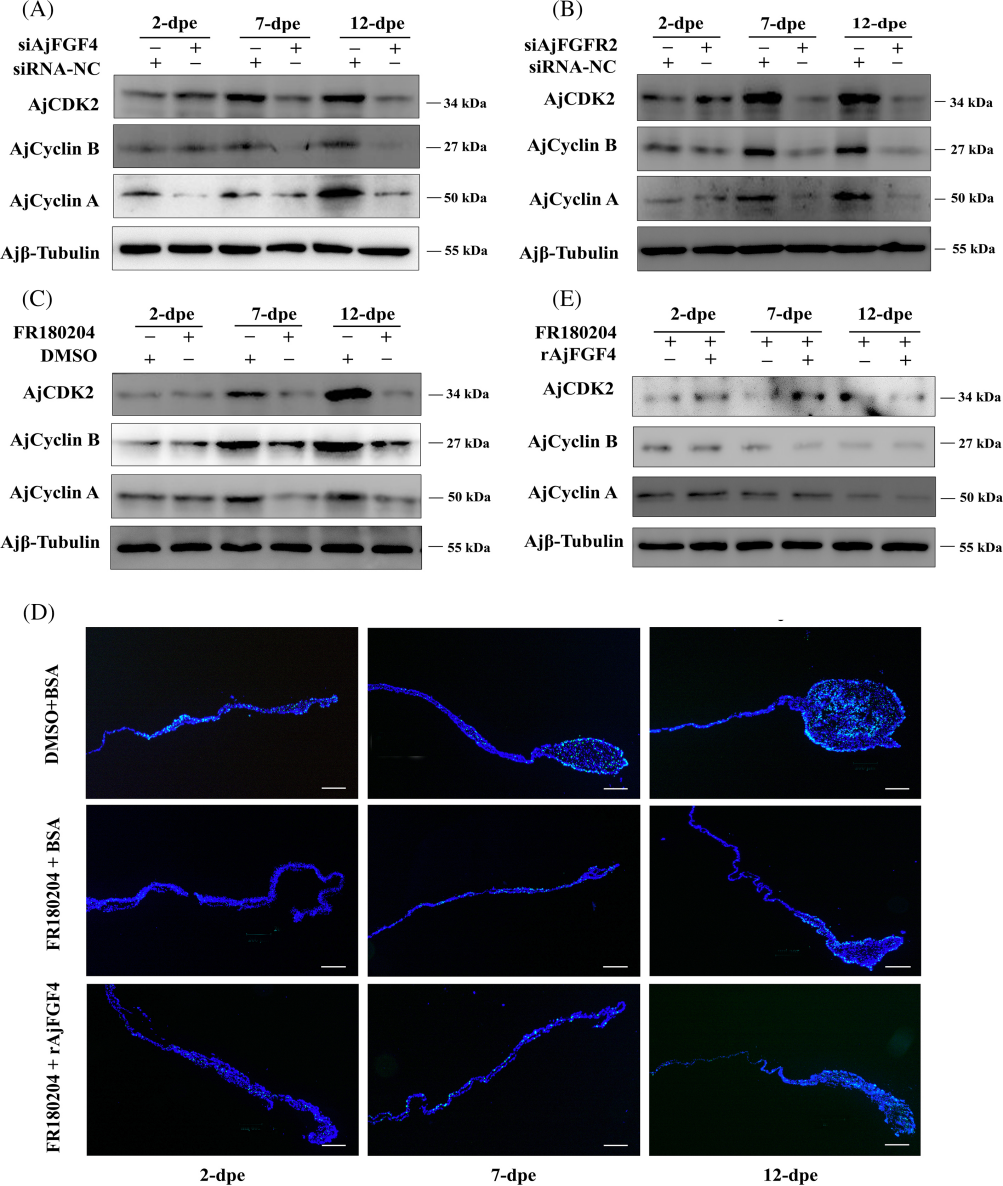
AjFGF4/AjFGFR2‐ERK–MAPK induced the expression of cell cycle‐related proteins (CDK2, Cyclin A, and Cyclin B) during intestine regeneration in *Apostichopus japonicus*. The protein expression levels of CDK2, Cyclin A, and Cyclin B in the regenerating mesentery and intestine at the stage of 2, 7, and 12 dpe were determined after siAjFGF4 (A), siAjFGFR2 (B), FR180204 (C), or FR180204 + rAjFGF4 (E) were analyzed by western blotting using anti‐CDK2 Ab, anti‐Cyclin B Ab, anti‐Cyclin A Ab, and anti‐Ajβ‐Tubulin Ab as the internal reference. Sea cucumbers were transfected with siRNA‐NC, siAjFGF4, siAjFGFR2, FR180204, DMSO, or FR180204 + rAjFGF4 in eviscerated‐sea cucumbers for six times in every 2 days. (D) EdU assay detection of cell proliferation and the size of regenerative tissues in the groups of FR180204 + BSA, FR180204 + rAjFGF4, and DMSO +BSA at 2, 7, and 12 dpe. The regeneration tissues in each group were taken from the same location in the anterior end (next to the esophagus) of the sea cucumber. Blue color indicates Hoechst‐stained nuclei, and green indicates EdU‐stained proliferating cells. Bar = 200 μm. In each experiment, the sample of six *A. japonicus* were analyzed and each experiment was repeated at least three times.

In mammals, the complex of cyclins and CDKs could drive cell from one stage to another during cell proliferation.^31^ In this study, to verify whether cell cycle‐related proteins were regulated by the AjFGF4/AjFGFR2‐ERK axis in regulating cell proliferation during intestine regeneration, we detected the protein levels of several cell cycle‐related marker proteins including CDK2, Cyclin A, and Cyclin B in eviscerated‐sea cucumbers post‐treated with siAjFGF4, siAjFGFR2, or FR180204. From the results of siRNA‐NC groups at different regeneration stages, it can be seen that the protein expressions of CDK2, Cyclin A, and Cyclin B in the mesentery and the newly formed intestinal rudiment were significantly increased with the progress of intestinal regeneration. However, they were significantly decreased at the stage of 2, 7, and 12 dpe after being treated with siAjFGF4 or siAjFGFR2 (Figure 7A,B). In line with this result, the protein expression levels of these three proteins were also much reduced at the stage of 2, 7, and 12 dpe in sea cucumbers treated with FR180204, relative to that in sea cucumbers treated with DMSO (Figure 7C). To further address whether the cell proliferation could be regulated by the AjFGF4/AjFGFR2‐ERK axis through the expression of cell cycle‐related proteins, we repeated FR180204 injected into eviscerated‐sea cucumbers for six times in every 2 days, and then supplemented it with the same dose of rAjFGF4 or BSA at 24 h post‐rAjFGF4 treatment to detect the changes in cell proliferation by the EdU staining assay, and the protein expression of CDK2, Cyclin A, and Cyclin B with immunoblot analysis. As expected, the size of regenerative tissues between the sea cucumbers treated with FR180204 + BSA and FR180204 + rAjFGF4 was not significant (Figures 7D and S12). Consistent with this result, we detected that the protein expression level of CDK2, Cyclin A, and Cyclin B in the FR180204 + rAjFGF4‐treated sea cucumbers showed no obvious changes compared with that in the FR180204 + BSA‐treated sea cucumbers under the same conditions (Figure 7E). In general, our results strongly indicated that AjFGF4/AjFGFR2–ERK pathway did activate the expression of cell cycle‐related proteins to induce cell proliferation during intestinal regeneration.

## 4 DISCUSSION

**FIGURE 8:**
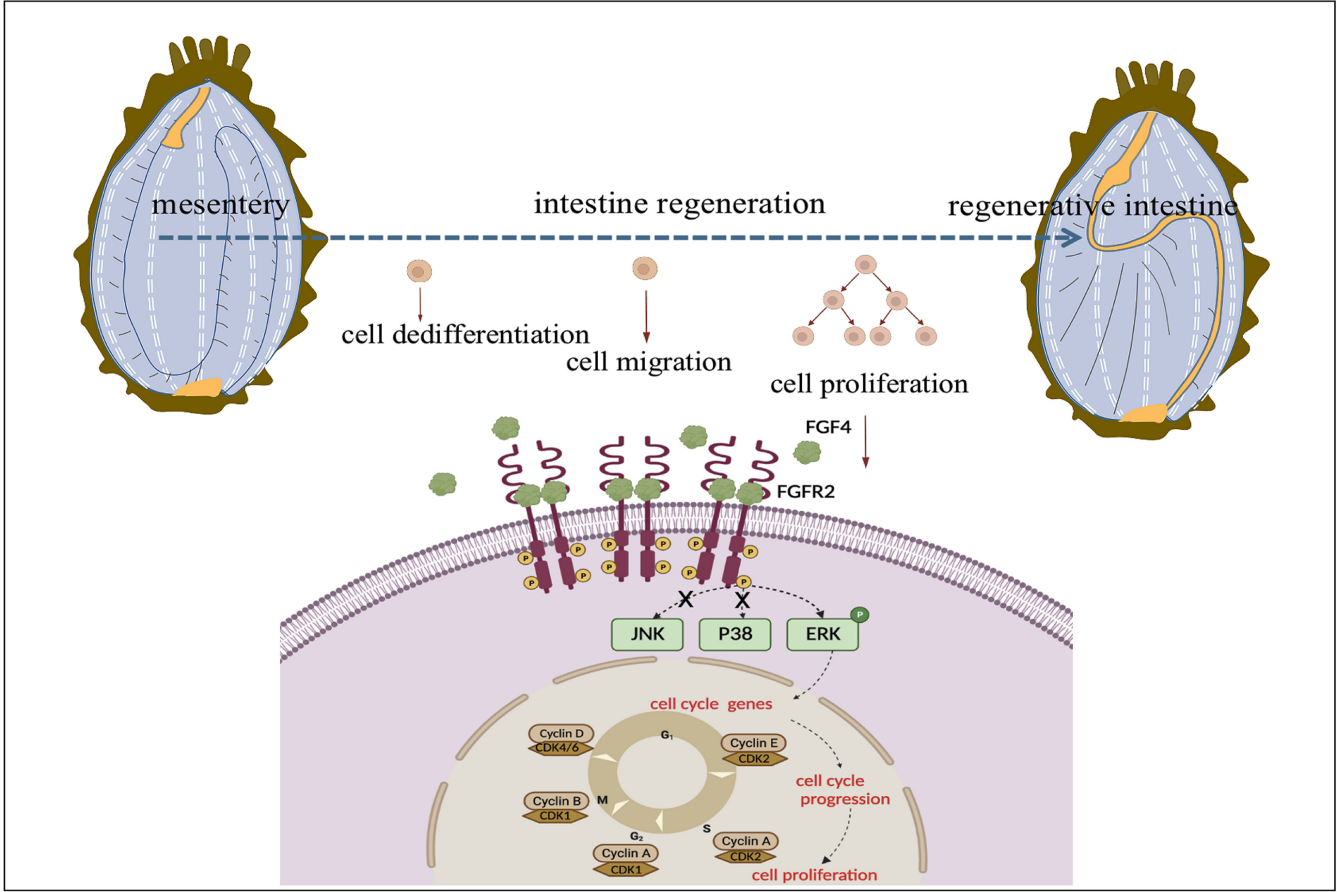
Scheme depicting how mesentery gradually thickened to form a new intestine and established a close relationship with cell proliferation. Furthermore, it illustrates how AjFGF4 induces cell proliferation in the process of intestinal regeneration. AjFGF4 associates with AjFGFR2 to stimulate cell proliferation through ERK activation, and then activated the expression of cell cycle‐related proteins, cyclins, and CDKs, to increase cell cycle progression, which finally promotes cell proliferation.

The ability to regenerate missing body parts is an awe‐inspiring natural phenomenon and also a major subject of medical research. While most animals can repair epidermal wounds, the capacity to regenerate missing body parts is restricted to few animals.^32^ Among these investigated regenerative species, echinoderm is one of the major groups of deuterostomes that can quickly regrowth most lost tissues/organs. Particularly, members of the class Holothuria can regenerate their intestine after a classic visceral resection. This new viscus formed in adult organisms provides a unique model for studying the process of organogenesis. Although several studies have been done on various aspects of sea cucumber regeneration, few studies have been carried out on the molecular mechanisms of intestinal regeneration post‐evisceration in sea cucumbers (*A. japonicus*).^33^ In the present study, we initially delineated the different stages of intestinal regeneration at the cellular and histological levels and provided the evidence that the swelling of the free end of the mesentery was necessary for the formation of the new intestine, which indicated that the mesentery was the epicenter for intestinal regeneration in *A. japonicus*. Notably, an increase in cellular proliferation was also observed closely with the size of the initial intestinal rudiment on the edge of the mesentery. Mechanically, we reported mesentery AjFGF4‐AjFGFR2‐ERK axis was involved in regulating intestinal regeneration via targeting cell cycle proteins in echinoderms. We found that AjFGF4 regulated *A. japonicus* cell proliferation depending on the recognition of AjFGFR2 during intestinal regeneration. Moreover, AjFGF4/AjFGFR2 modulated intestinal regeneration by promoting the expression of the cell cycle‐related proteins (CDK2, Cyclin A, Cyclin B) by specifically activating the ERK–MAPK pathway, but not the JNK and the p38 pathway (Figure 8). This finding was of great significance for further exploring the molecular regulatory mechanism in regulating the development of intestinal regeneration in echinoderms and even mammals.

The experiments described in this study established the fact that the excised *A. japonicus* intestine after spontaneous evisceration was replaced by a regenerative process that likely followed a pattern of morphological changes and cell fate changes consistent with other sea cucumber species.^17^, ^18^ As pointed out by Okada and Kondo,^33^ despite the differences in type of spontaneous evisceration from the anterior or posterior ends of the body for different species of sea cucumbers, the mesentery which connected the internal lining of the body cavity and the digestive tract were evidently of great significance in the regeneration of new intestine. However, in the process of intestine regeneration in *A. japonicus*, previous studies had paid more attention to the formation of the intestinal cavity and the comparisons between regenerating and normal intestine, but less on the role of the mesentery in the whole stage of intestinal regeneration.^34^ In this study, we found that the regeneration of intestine always involved an initial thickening of the free edge of the mesentery, which enabled the lumen of the new intestine forms. Such close relationship between regenerating intestine and the remnants of the mesentery of *A. japonicus* was parallel with the situation previously described in visceral regeneration of various sea cucumbers.^17^, ^35^, ^36^ These studies provided strong evidence that the mesentery of sea cucumber played an important role in intestinal regeneration. However, due to the technique limitation, we have not been able to directly explore the contribution of the mesentery in the process of intestinal regeneration by removing the mesentery from sea cucumbers in vivo. In addition, the role of the mesentery in regeneration of the digestive tract had not been well studied in other animals, even though this tissue arrangement between the mesentery and intestine of sea cucumber usually follows the pattern characteristic of other coelomic animals.^18^ The issue of whether the mesentery played a role in the regenerative phenomenon in other animals, or whether it could be induced to play such a role is open to future investigation. Furthermore, the thickening of the mesenteric terminal was similar to the formation of the thickened blastema following amputation of a urodele limb or teleost fin, which was a crucial step in facilitating subsequent regeneration. The research on the mechanism of regeneration primordium thickening in these regeneration processes could provide a reference for regeneration research.

Crucial to the understanding of organ regeneration was identifying the origin of the cells that form the new regenerated structure which organized by a series of events including cell dedifferentiation, cell migration, cell proliferation, cell differentiation, and morphogenesis.^37^ Experimental results had shown some similarities and differences among regenerating animal groups. For example, in planarians, regeneration depended on a population of proliferating stem cells, that could give rise to all cell phenotypes.^38^ In contrast, cells adjacent to the injury dedifferentiate, proliferate, and then gave rise to the cells that regenerating in zebrafish heart regeneration.^39^ In our case, during the process of intestine regeneration of *A. japonicus*, we observed myofibroblastc dedifferentiation (Figure 1) and a large number of active cell proliferation during intestinal regeneration, which might explain the cell source of the thickening of the mesentery to form the new intestine. In the early stage of regeneration, the cells of mesentery undergo dedifferentiation and return to the original state, that once dedifferentiated they proliferated for a short time to form a primordium, which provided the basis for subsequent cell differentiation to provide various types of cells required for intestinal regeneration mass. In addition, during regeneration, cell proliferation was tightly regulated in space and time, and was crucial to the success of regeneration.^40^ In the *A. japonicus* intestinal system, our study found that the cell proliferation took place primarily in the mesothelial layer and luminal epithelium of the distal mesenterial area. It began slowly during the first 2 dpe while increasing gradually with the thickening of the intestinal rudiment from the 7 dpe. The continuous cell proliferation in mesentery supplied additional cells necessary for the increase in the size of these thickened regeneration primordia and underlies the formation of a new intestine. The mitotic rate of the mesentery especially in the mesenterial tip would determine the development of regeneration.

Regeneration was controlled by a variety of cytokines, growth, and differentiation factors. Among them, FGFs constituted a family of conserved small proteins playing crucial roles during the process of both organogenesis and tissue homeostasis after injury. From an evolutionary perspective, the FGF gene family was involved in extensive gene expansion and gene contraction in different species, often lineage specific,^41^ resulting in a complex and variable distribution of FGF genes in metazoans. In vertebrates, gene expansion of the gene family occurred resulting in 27 FGFs in zebrafish and over 22 FGFs in mammals.^42^, ^43^ However, FGF gene families contracted significantly in echinoderms. It is reported that only one FGF8 gene exists in *A. japonicus*, *S. purpuratus*, and *A. planci* through early genomic analysis.^44^ In this study, we obtained another FGF in the transcriptional data analysis of *A. japonicus* intestinal regeneration, named AjFGF4 by sequence alignment and phylogenetic tree analysis. Through subsequent gene interference experiments, we confirmed the regulatory role of AjFGF4 on the proliferation of mesenteric and regenerative primordial cells during intestinal regeneration. The importance of FGFs in tissue regeneration had been confirmed in species with high regeneration ability, such as amphibians and fish, as well as in the few cases in which mammals also regenerate lost tissues.^27^ In higher mammalian studies, the signal transduction of FGFs relies on the binding and activation of four transmembrane tyrosine kinase receptors, named FGFR1‐4, and the complexity of FGFRs is achieved through alternative splicing.^45^ Of particular importance was alternative splicing of the RNA encoding the third immunoglobulin‐like domain (Ig III) of FGFRs 1, 2, and 3, which generates the IIIb and IIIc variants, which differ in their ligand‐binding specificities.^45^ These isoforms were usually differentially expressed in the epithelium or mesenchyme and display different ligand binding capabilities. In this study, we predicted the binding of AjFGF4 to four FGFRs by molecular docking and the result showed that AjFGFR2 binds to AjFGF4 with more non‐covalent bonds compared to AjFGFR1, AjFGFR3, and AjFGFR4, and it was speculated that AjFGFR2 had the highest probability of combining with AjFGF4. However, despite the further confirmation of the binding between AjFGF4 and AjFGFR2 by Co‐IP assay, GST‐pull down, and laser confocal microscopy assay, it could not exclude the possibility of AjFGF4 binding to the other three AjFGFRs, which might be the directions in our future research.

FGFs were originally discovered due to their ability to induce fibroblasts proliferation, and the data from tissue regeneration, embryogenesis, and cancer also supported the role of FGF signaling in promoting the proliferation of cells and tissues other than fibroblasts.^27^, ^42^ Since then, FGF signaling had been shown to function through activating various signaling cascades, of which the MAPK pathway was the most prominent, to regulate a variety of cellular processes, including cell proliferation and cell migration.^31^ MAPKs consisted of three principal family members (ERKs, p38, and JNKs) that activated different pathways in different upstream activation responses or in different tissue types.^1^ For example, in mammalian cardiac myocytes, the ERK cascade was thought to be primarily activated in response totyrosine kinase receptor and G protein‐coupled receptor (GPCR) activation, while the p38 and JNK cascades were activated by both GPCR activation and stress signals.^46^ In the sea cucumber model, our data showed that AjFGF4/AjFGFR2 had no effect on the p38‐MAPK and JNK–MAPK signaling pathway to regulate cell proliferation during intestinal regeneration. In the ERK–MAPK pathway, AjFGF4/AjFGFR2 could only induce the phosphorylation levels of ERK, but not the protein levels under the same conditions. The MAPK/ERK inhibitor assay further demonstrated that AjFGF4/AjFGFR2 activated the ERK1/2 pathway in eviscerated sea cucumbers. Many studies had also shown that ERK1/2 functioned as a common mediator of FGF signaling in promoting cell proliferation in *Xenopus laevis*, zebrafish, and axolotl.^47^, ^48^ Interestingly, we further identified AjERK2 acted as the likely mediator of FGF signaling in modulating cell proliferation based on its silencing and/or following AjFGF4 treatment in cultured single‐cell of the mesentery at the stage of 7 dpe in vitro. Taken together, these findings suggested that AjFGF4/AjFGFR2 regulated cell proliferation via activating the ERK1/2 pathway during intestinal regeneration in eviscerated sea cucumbers.

Cell proliferation was a critical requirement for tissue regeneration, and regulation of cell cycle entry and progression was particularly important for this process.^49^ In the process of cell proliferation, the complex of cyclins and CDKs could facilitate cells from one stage to another.^50^ On the contrary, an abnormally regulated cell cycle might turn normal cells into cancer cells. In the present study, the protein expression levels of CDK2, Cyclin A, and Cyclin B were much upregulated at the 2, 7, and 12 dpe in sea cucumbers, which was consistent with the results of the increasing trends of cell proliferation under the same conditions. Many studies had also shown that FGF and/or alternative molecules could induce cell cycle‐related proteins to regulate cell progress and proliferation in different normal and cancer cells. In this study, we found that the expression levels of these three proteins were significantly decreased post‐siAjFGF4, siAjFGFR2, or ERK inhibitors treatment. Furthermore, cultured cells treated with rAjFGF4 + ERK inhibitor showed no significant difference in the cell proliferation levels compared with ERK inhibitor‐treated cells. Thus, our results strongly showed that AjFGF4/AjFGFR2 activated cell proliferation by targeting cell cycle‐related proteins through the ERK–MAPK pathways during intestinal regeneration.

In summary, our study showed that the mesentery was the epicenter for intestinal regeneration in *A. japonicus*. These findings also provided a shred of direct evidence that the increased cell proliferation was positively correlated with the size of initial intestinal rudiment in the edge of the mesentery. Mechanistically, AjFGF4 bound its receptor, AjFGFR2, and then activated ERK–MAPK pathway by inducing ERK phosphorylation and upregulating the expression of cell cycle‐related proteins to induce cell proliferation during intestinal regeneration in *A. japonicus*. In this study, we used an *A. japonicus* model for the first time to provide functional evidence that intestinal regeneration was regulated through the mesentery AjFGF4–AjFGFR2–AjERK pathway by targeting the cell cycle in echinoderms. These findings might offer new insights into the understanding of tissue regeneration and also help establish treatments to drive regeneration rather than repair processes in poorly regenerating organisms, including humans.

## AUTHOR CONTRIBUTIONS

Chuili Zeng performed the experiments, interpreted the data, and wrote the manuscript. Chenghua Li participated in the experimental design, interpreted the data, contributed new reagents, analytic tools, and revised the manuscript. Ming Guo revised the manuscript. Yangxi Xiang, Mingshan Song, and Ke Xiao performed the partial experiment. All authors read and approved the final version of the manuscript.

## FUNDING INFORMATION

This work was supported by the National Natural Science Foundation of China (32073003), Natural Science Foundation of Zhejiang Province (LZ19C190001), Research and Innovation Fund of Ningbo University (IF2022151) and the K.C.Wong Magna Fund in Ningbo University to Chenghua Li. The funders had no role in study design, data collection and analysis, decision to publish, or preparation of the manuscript.

## CONFLICT OF INTEREST

The authors declare no conflict of interest.

## Supporting information


**Data S1** Supporting information

## Data Availability

All study data are included in the article and/or supporting information. All the numerical data can be found in the Source Data Excel files associated with each figure.
