# Supplementary material for: Mesentery AjFGF4–AjFGFR2–ERK pathway modulates intestinal regeneration via targeting cell cycle in echinoderms
Source: Cell Prolif. 2022 Oct 20;56(2):e13351. doi: 10.1111/cpr.13351 (PMC9890533; doi:10.1111/cpr.13351)
Supplement: Supplementary file 1 — Data S1 Supporting information [file CPR-56-e13351-s001.docx]

**Supplementary materials**

**
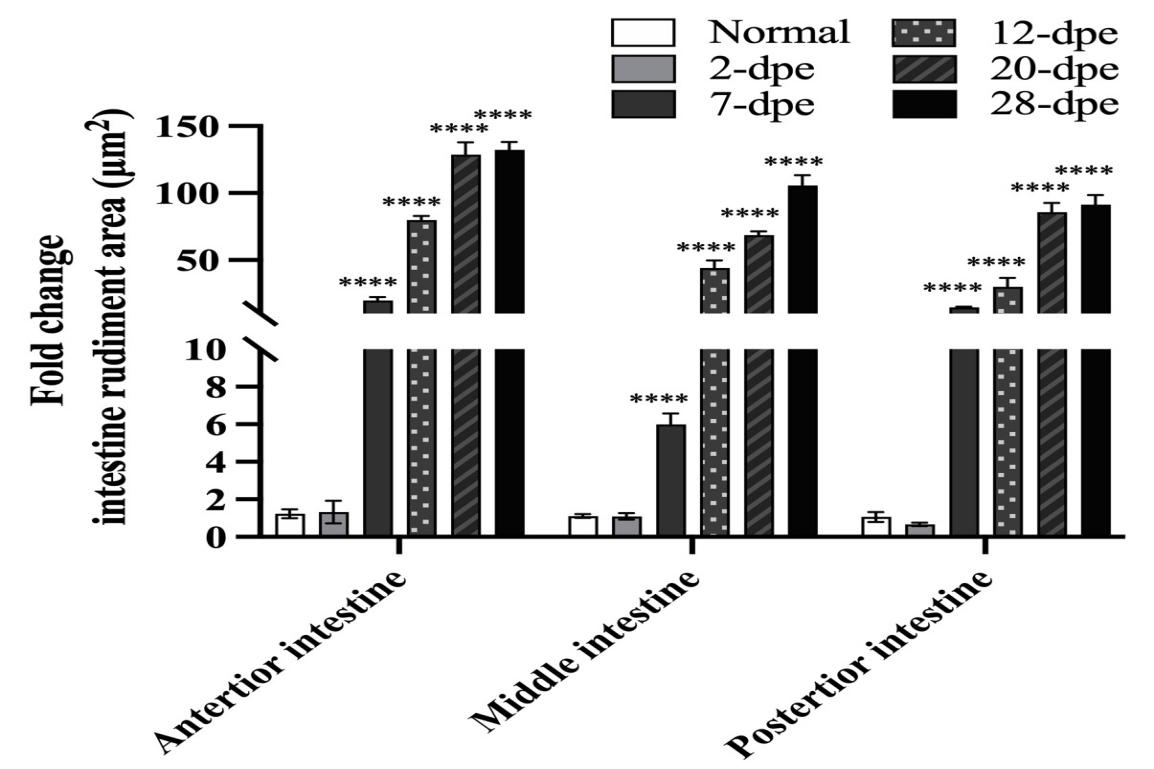
**

**Figure supplement 1 for Figure 2.** The size of the intestine rudiment area was measured at normal and 2-dpe, 7-dpe, 12-dpe, 20-dpe, 28-dpe stage by the Image J software (<http://rsbweb.nih.gov/ij/).> Data are the means of three independent experiments and are presented as the mean ± SD, *****p* < 0.0001.


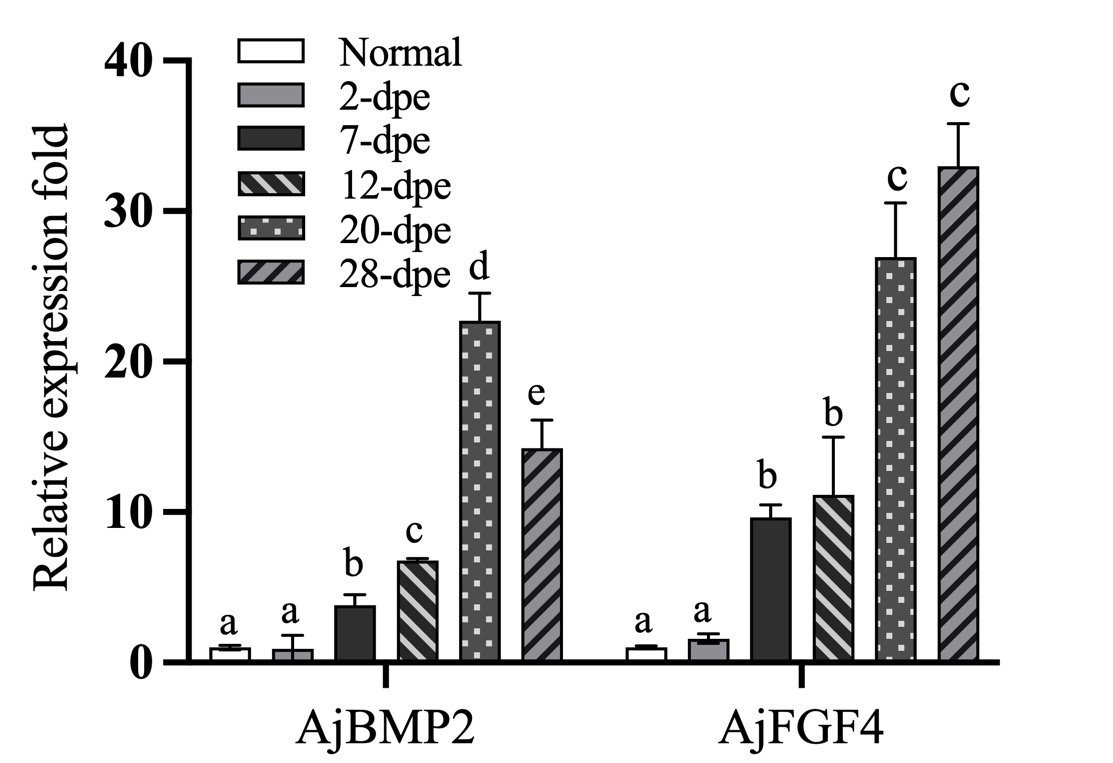


**Figure supplement 2 for Figure 3.** Relative expression of AjBMP2 and AjFGF4 in the normal and different stages of intestinal regeneration.


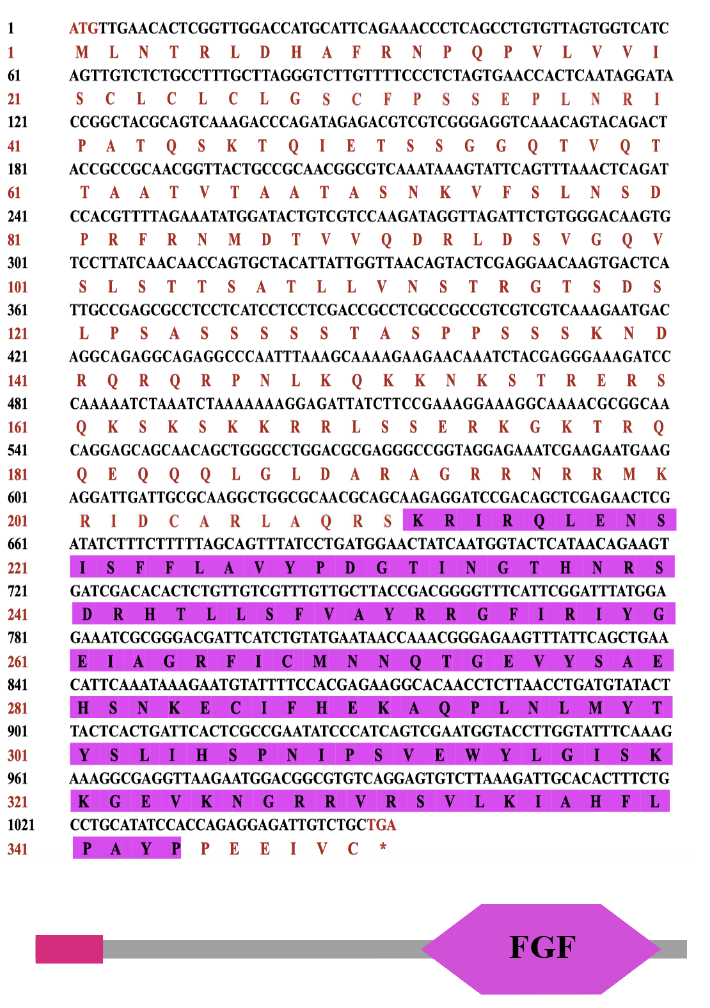


**Figure supplement 3 for Figure 3.** The nucleotide, amino acid sequences and the domain analysis of AjFGF4.


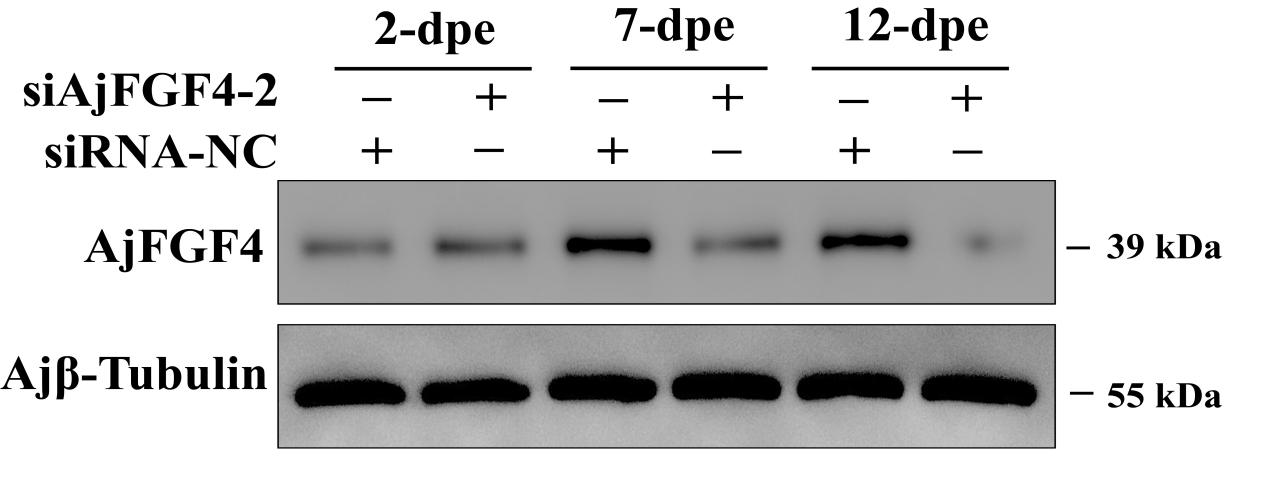


**Figure supplement 4 for Figure 3.** The protein levels of AjFGF4 in regenerating mesentery and intestine at 2-, 7- and 12-dpe stage after siRNA-NC or siAjFGF4-2 injection.

**
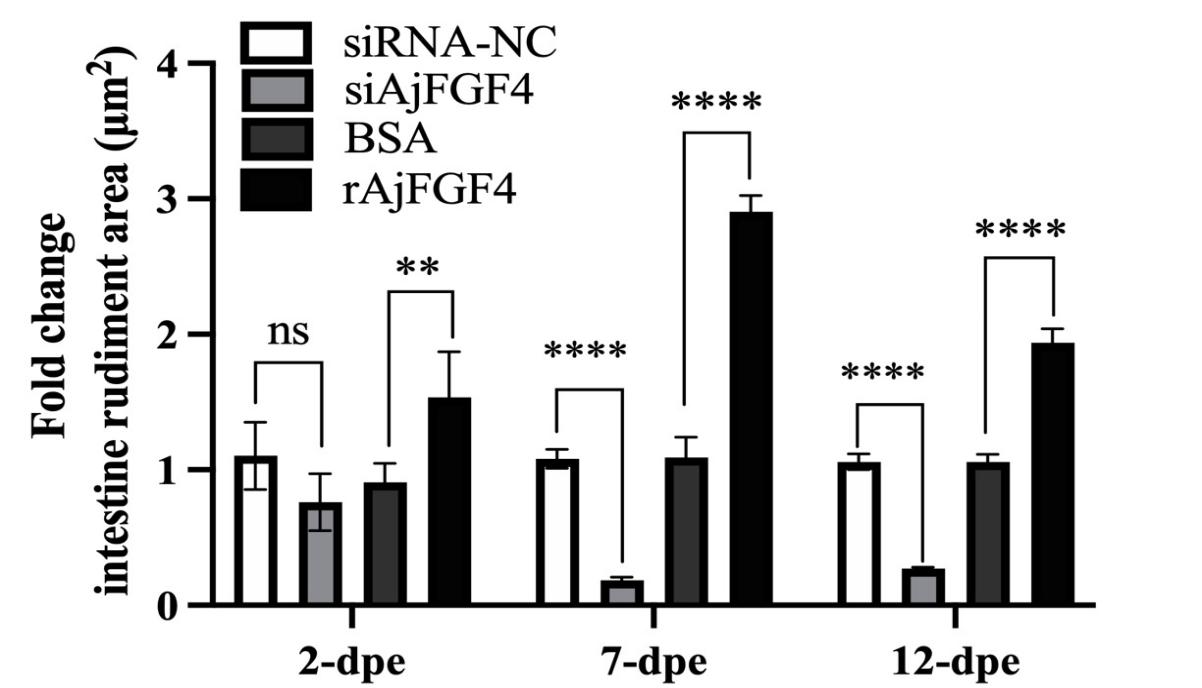
**

**Figure supplement 5 for Figure 3.** The size of the intestine rudiment area was measured at siRNA-NC, siAjFGF4, BSA, rAjFGF4 group at 2-dpe, 7-dpe, 12-dpe stage. Data are the means of three independent experiments and are presented as the mean ± SD, *****p* < 0.0001.

**
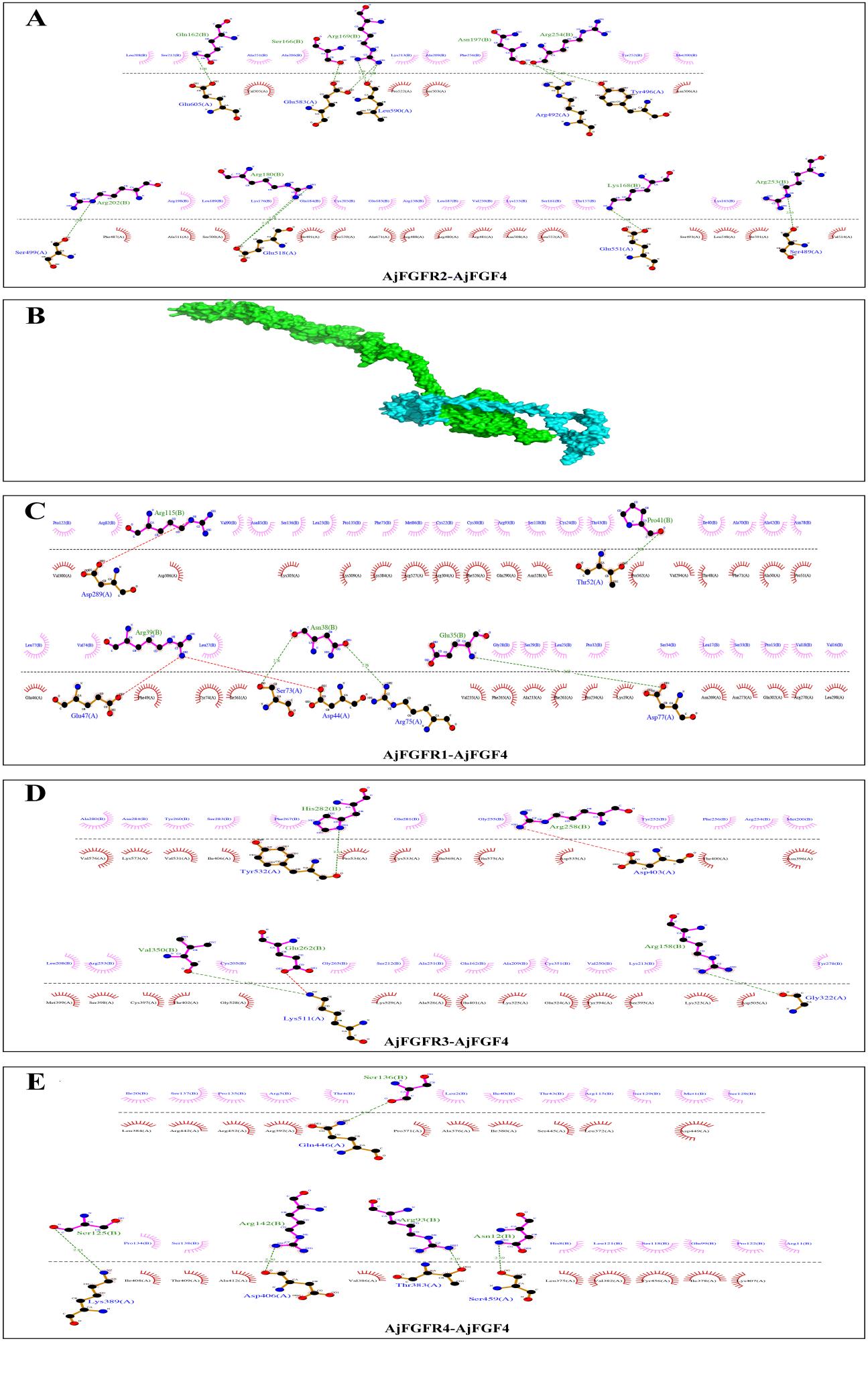
**

**Figure supplement 6 for Figure 4.** The FGFR family of sea cucumber consists of 4 receptor genes, namely AjFGFR1 to AjFGFR4. Global docking using the ZDOCK server predicted that AjFGF4 interacted with AjFGFR1-4 and drawled a 2D docking diagram. AjFGFR2 (**A**), AjFGFR1 (**C**), AjFGFR3 (**D**), AjFGFR4 (**E**). Green broken line: H-bond between the AjFGF4 and AjFGFR1-4. (**B**) The 3D diagram of molecular docking between AjFGF4 and AjFGFR2 were predicted by PyMOL program. The green structure represents AjFGF4 and the blue structure represents AjFGFR2.


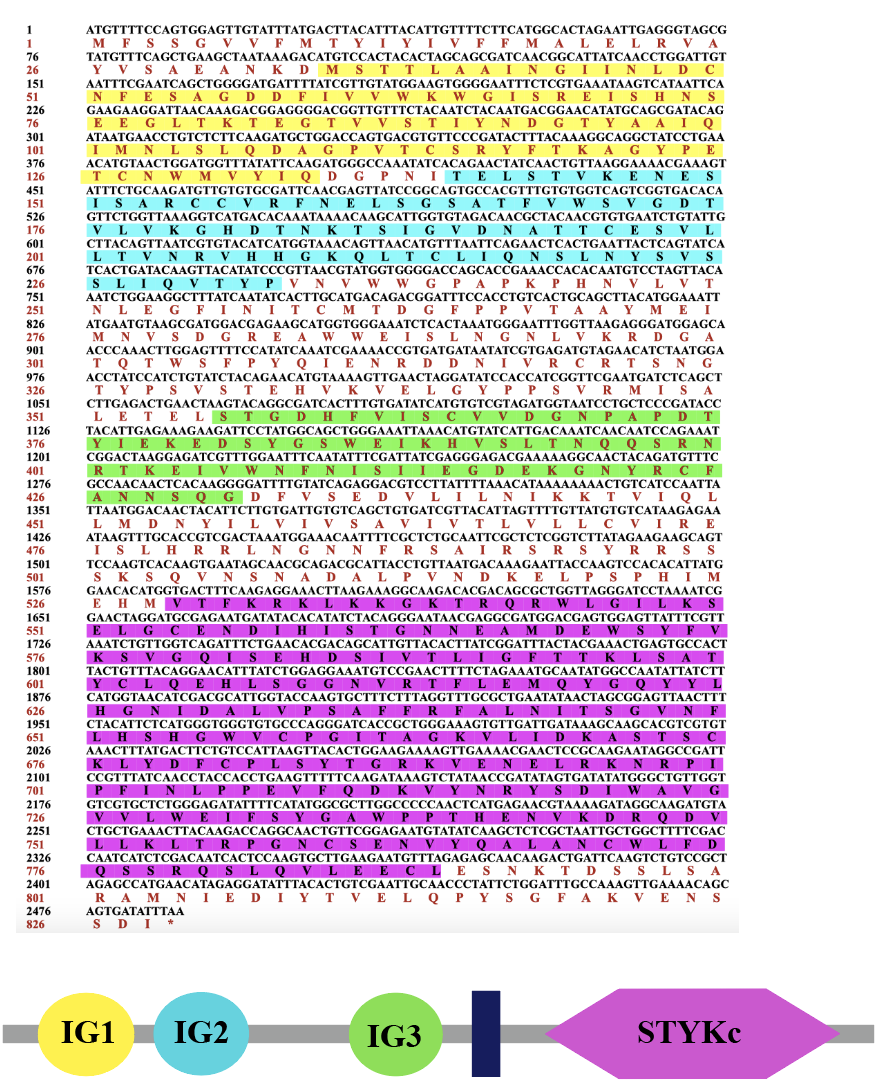


**Figure supplement 7 for Figure 4.** The nucleotide, amino acid sequences and the domain analysis of AjFGFR2.


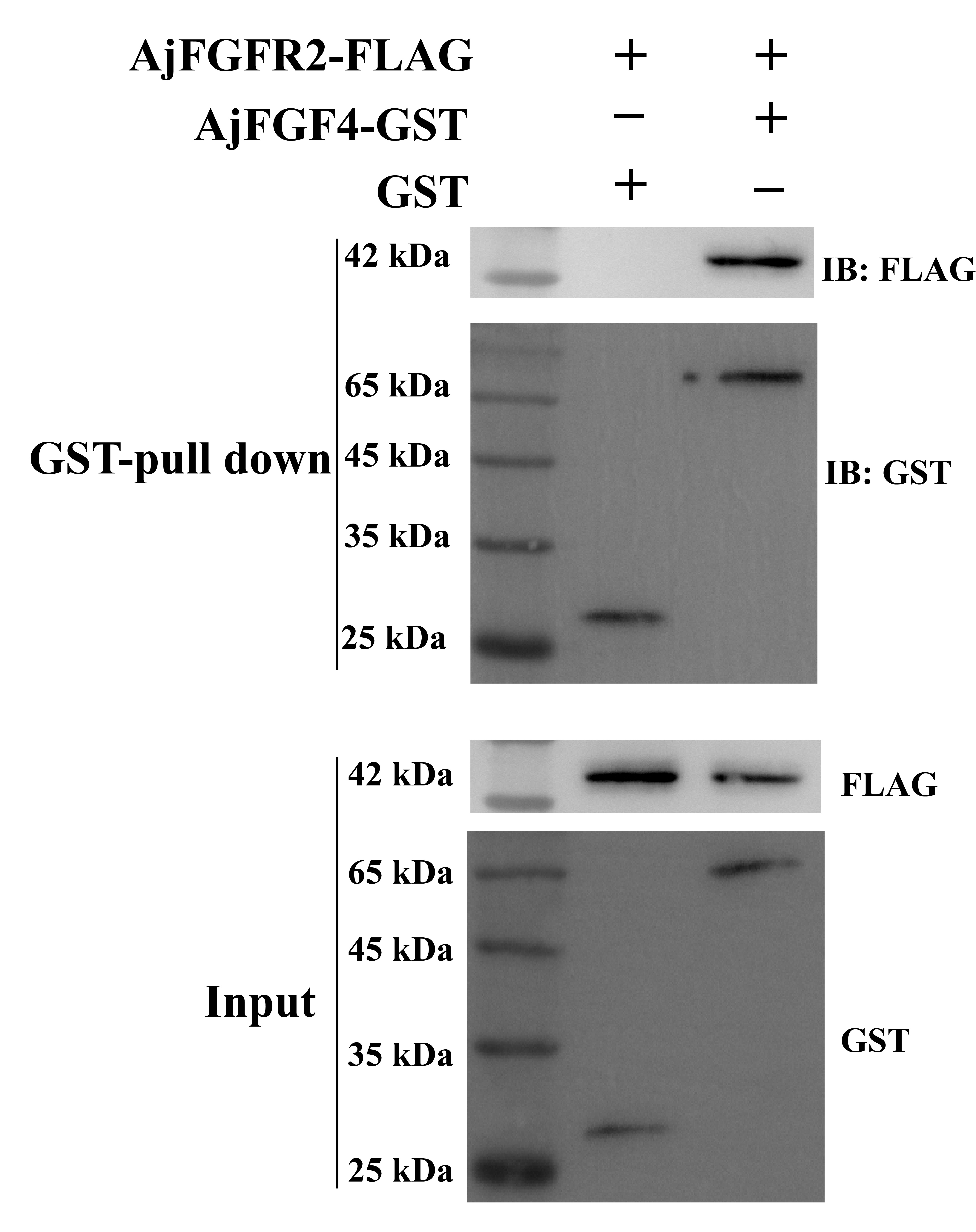


**Figure supplement 8 for Figure 4.** Interactions between AjFGFR2-flag and AjFGF4-GST were detected by pull-down assays. FGFR2-flag or empty vector (5 μg) transfected HEK293T cells were lysed and incubated with GST-tag labeled AjFGF4 or GST-tag protein and detected by western blotting using the anti-flag Abs.


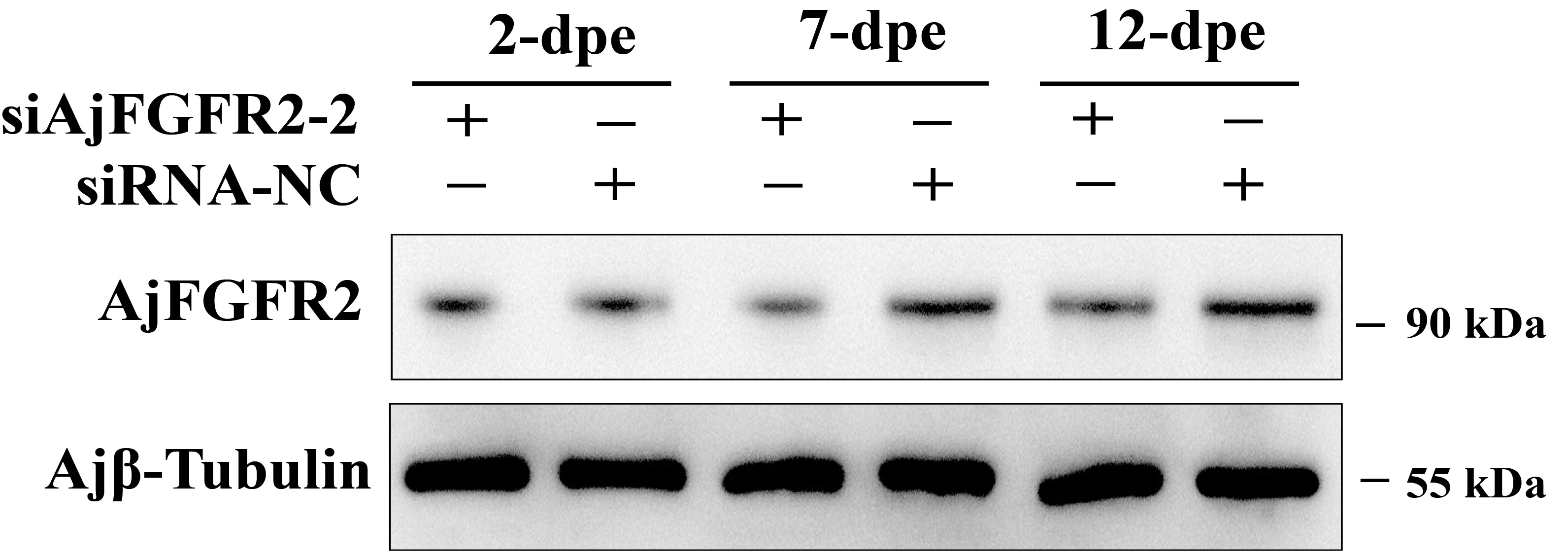


**Figure supplement 9 for Figure 5.** The protein levels of AjFGFR2 in regenerating mesentery and intestine at 2-, 7- and 12-dpe after siNC or siAjFGFR2-2 injection.


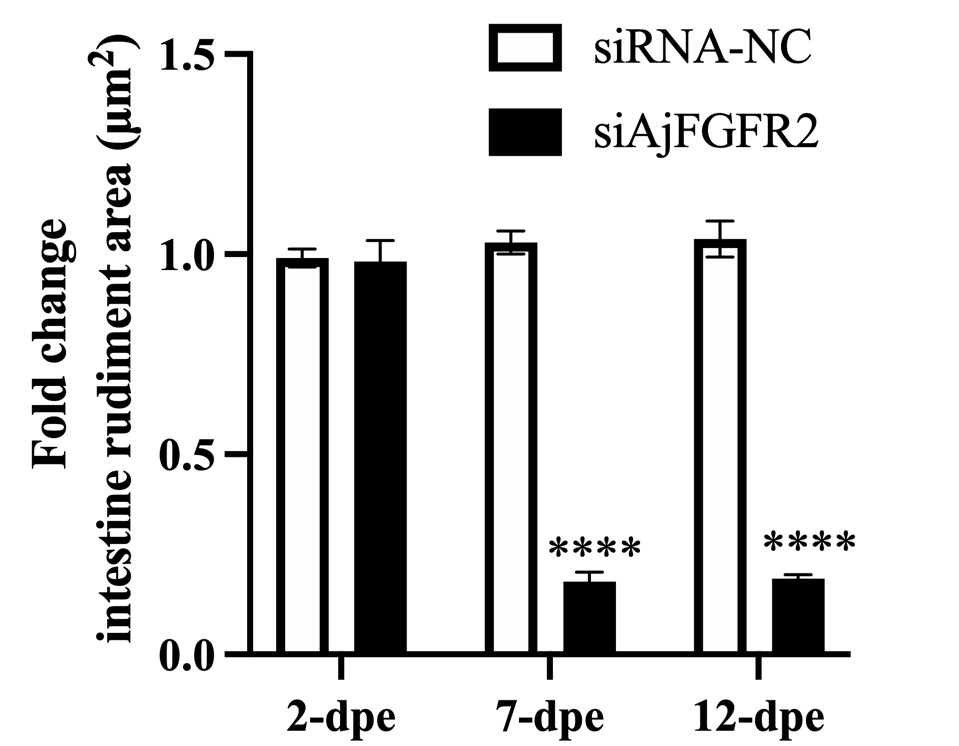


**Figure supplement 10 for Figure 5.** The size of the intestine rudiment area was measured at siRNA-NC and siAjFGFR2 group at 2-dpe, 7-dpe, 12-dpe stage. Data are the means of three independent experiments and are presented as the mean ± SD, *****p* < 0.0001.


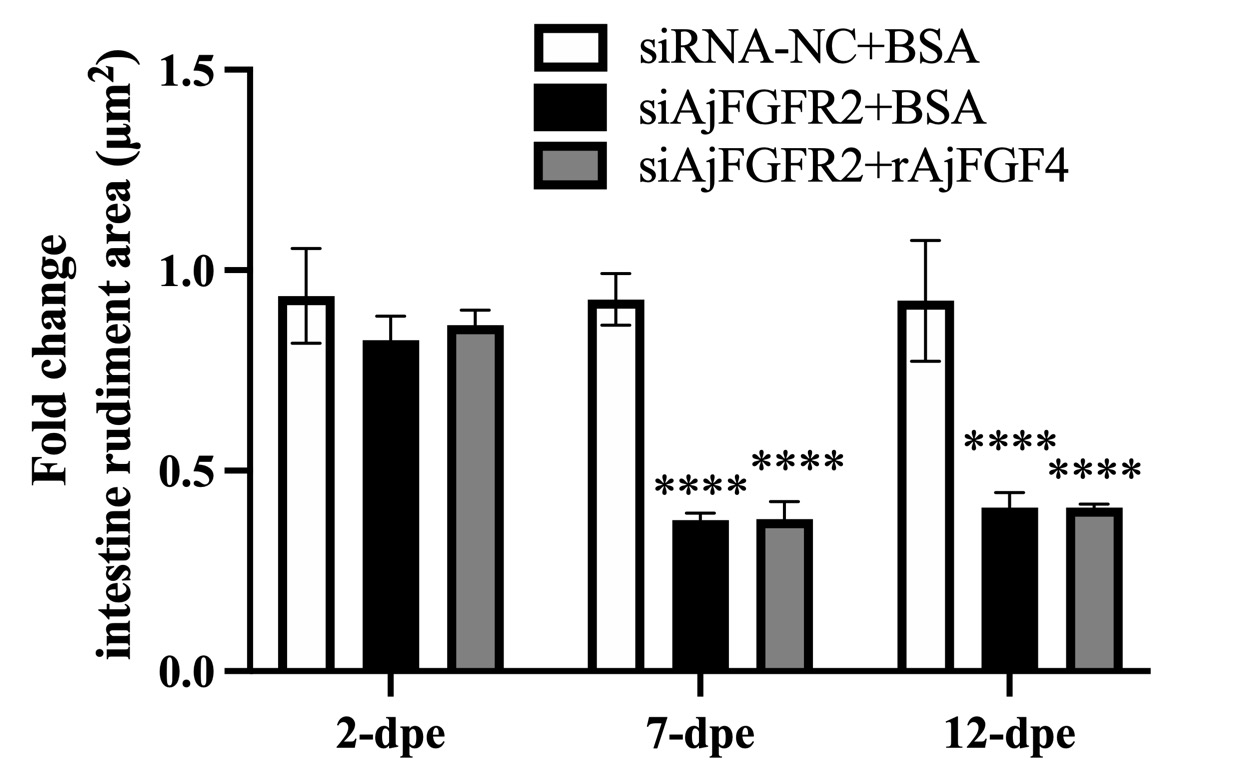


**Figure supplement 11 for Figure 5.** The size of the intestine rudiment area was measured at siRNA-NC + BSA, siAjFGFR2 + BSA and siAjFGFR2 + rAjFGF4 group at 2-dpe, 7-dpe, 12-dpe stage. Data are the means of three independent experiments and are presented as the mean ± SD, *****p* < 0.0001.


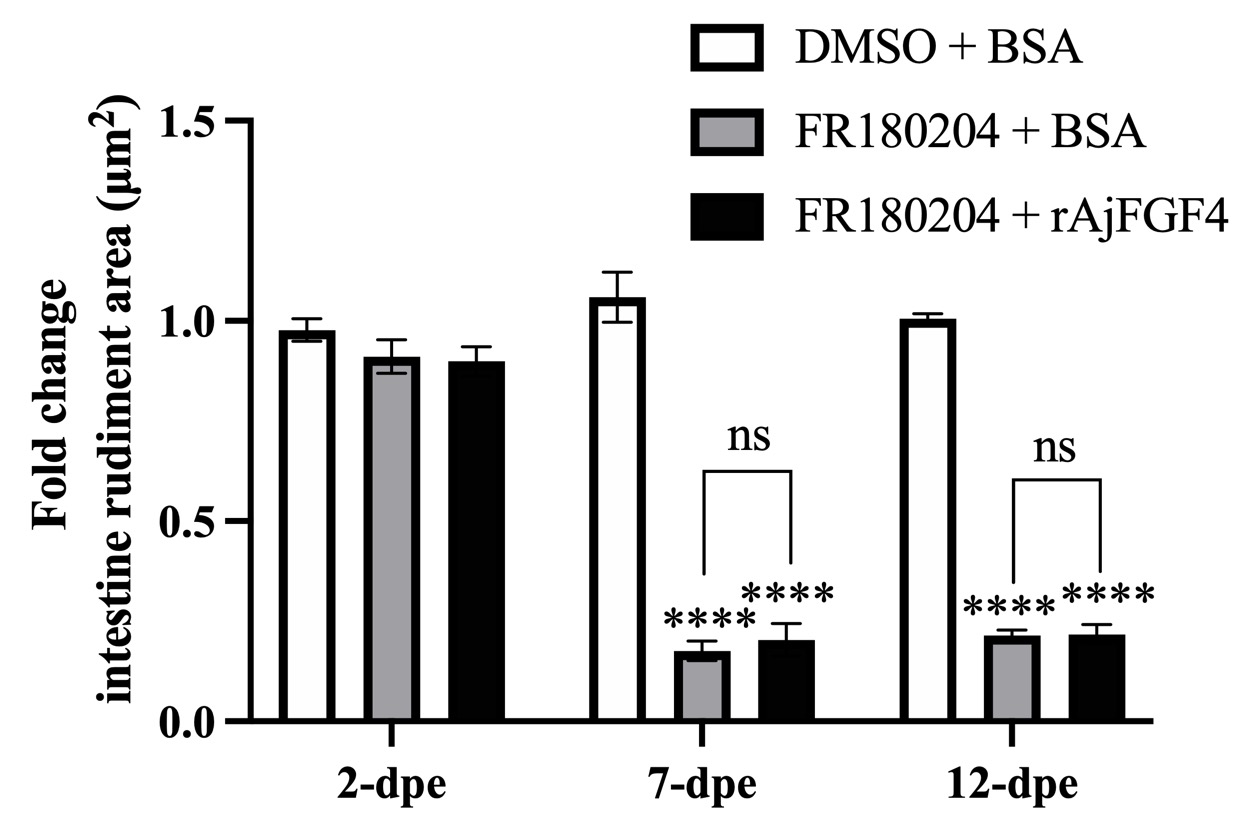


**Figure supplement 12 for Figure 7.** The size of the intestine rudiment area was measured at DMSO + BSA, FR180204 + BSA and FR180204 + rAjFGF4 group at 2-dpe, 7-dpe, 12-dpe stage. Data are the means of three independent experiments and are presented as the mean ± SD, *****p* < 0.0001.
